# Programmable pH-responsive DNA inter-strand matching (PRISM) for precision molecular band-pass actuation

**DOI:** 10.7150/thno.136316

**Published:** 2026-06-17

**Authors:** Xiaole Han, Hongyan Yu, Xiaomei Lin, Li Zhang, Weitao Wang, Yaoyi Zhang, Jianbo Jiang, Xingyu Liu, Ke Lv, Guoming Xie

**Affiliations:** 1 Department of Neurosurgery, Laboratory of Neurological Diseases and Interdisciplinary Medicine, The First Affiliated Hospital of Chongqing Medical University, No.1 Youyi Road, Chongqing, 400016, China.; 2 Key Laboratory of Clinical Laboratory Diagnostics (Chinese Ministry of Education), College of Laboratory Medicine, Chongqing Medical University, Chongqing, 400016, China.

**Keywords:** pH-responsive, band-pass filter, tumor microenvironment, CRISPR/Cas13a, DNA nanotechnology

## Abstract

**Background:**

Precise sensing of narrow physiological pH ranges is important for accurate molecular regulation in complex biological environments. Traditional pH-responsive systems generally rely on monotonic off-to-on switching, making it difficult to distinguish subtle differences in tumor microenvironment pH (6.5-6.8) from neutral healthy tissues or highly acidic lysosomal compartments.

**Methods:**

We developed PRISM (pH-Responsive DNA Inter-Strand Matching), a universal and DNA-based strategy that functions as a programmable molecular band-pass filter. PRISM combines two antagonistic regulatory domains-an i-motif-mediated low-pH OFF module and a C-A mismatch-mediated high-pH OFF module-within a three strand framework. Through sequence-level thermodynamic design, the system defines a controllable operational window that enables non-monotonic molecular actuation.

**Results:**

Systematic characterization showed that PRISM generated band-pass signal responses with tunable operational range. The platform was successfully applied to high-contrast imaging of subtle pH gradients on HeLa cell surfaces, enabling effective discrimination of tumor microenvironment from both neutral and highly acidic environments. In addition, PRISM enabled allosteric regulation of fluorogenic DNA Lettuce aptamers and enabled logic gated control of CRISPR/Cas13a activity by integrating synthetic DNA modules with the intrinsic pH responsiveness of enzymes.

**Conclusion:**

Beyond conventional monotonic switching toward precise windowed regulation, PRISM offers a modular and biocompatible strategy for the development of intelligent theranostic platforms. This strategy facilitates high-fidelity diagnostic imaging and localized therapeutic activation within subtle pathological microenvironments without the need for complex bioconjugation.

## Introduction

The ability to recognize and respond to environmental signals is fundamental to both biological systems and engineered nanodevices 1-3. In nature, many biochemical processes, including enzymatic catalysis and antigen processing, occur only within limited physiological rather than changing monotonically with environmental conditions 4-6. Reproducing such windowed or band-pass responsiveness in synthetic systems is important for precision theranostics, where accurate diagnosis and localized molecular regulation are expected to function under complex biological conditions 7-9. Among environmental factors, pH is widely used as an important biological indicator 10-12. For example, tumor tissues often exhibit mildly acidic extracellular conditions (pH 6.5-6.8) 13, 14, which differ from both the neutral physiological pH (7.4) of healthy tissues and acidic lysosomal environments (pH < 5.5) inside cells 15, 16. In clinical practice, difficulty in distinguishing these subtle pH differences may lead to false-positive diagnostic results or undesirable off-target therapeutic effects.

Most pH-responsive systems exhibit simple all-or-nothing switching and usually show monotonic acid-on or base-on responses 17-24. Although several approaches have been explored to address this issue, they still rely on chemically engineered materials. For example, studies have employed an “accelerator/brake” strategy in core-satellite nanoparticles to achieve tumor-selective reactive oxygen species (ROS) generation 25, or used cascade heterogeneous proton nanotransistors to construct BLINK polymers for multiplex pH-interval imaging 26. Despite their usefulness in tumor microenvironment imaging, systems based on synthetic polymers or inorganic hybrid materials may be less compatible with functional biological components, making combined diagnostic and therapeutic applications more difficult.

DNA nanotechnology provides another option because of its biocompatibility, predictable hybridization behavior, and sequence-dependent design flexibility 27-29. Different pH-responsive DNA motifs, including i-motifs and triplexes, have already been incorporated into biosensing systems 24, 30-34. However, their applications in generating non-monotonic pH responses remain limited. In our previous work, SHADE strategy was developed, demonstrating that chaperone-like auxiliary mechanisms can be used to construct narrow-window sensors 35. Nevertheless, it requires specific auxiliary components, leaving a gap in creating a general framework for direct biological execution. Achieving a purely DNA-based narrow-band pH-driven platform has remained a challenging goal. Unlike synthetic chemical polymers, pure DNA systems allow for modularity that enables the seamless integration of sensing logic with biological actuators without complex bioconjugation chemistry 36, 37.

**Figure 1:**
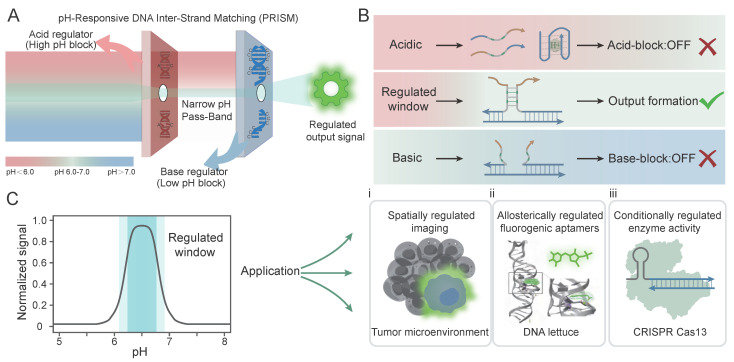
Design and mechanism of the pH-Responsive DNA Inter-Strand Matching (PRISM) strategy for programmable band-pass molecular actuation. (A) Conceptual overview of PRISM as a molecular filter. The system acts as a band-pass filter that selectively allows output signal formation within a narrow pH window, governed by synergistic Acid and Base regulators using C-A mismatches and i-motif sequence. (B) Molecular mechanism of the band-pass logic. PRISM integrates antagonistic regulatory domains within a three-strand hybridization framework. Under acidic conditions, i-motif formation triggers strand dissociation (Acid-block: OFF). Under basic conditions, the thermodynamic instability of C-A mismatches prevents core hybridization (Base-block: OFF). Stable hybridization and signal output (ON) are confined to the pre-programmed regulated window. (C) Performance and diverse bio-applications of PRISM. The plot shows a representative band-pass profile centered in the target window. The platform's modularity enables: (i) spatially regulated imaging of the tumor microenvironment; (ii) allosteric control of DNA Lettuce fluorogenic aptamers; and (iii) conditional regulation of CRISPR/Cas13a enzyme activity.

In this work, we present a DNA-based multifunctional strategy termed PRISM (**p**H-**R**esponsive DNA **I**nter-**S**trand **M**atching) for precision control of molecular actuation (Figure 1A). The platform utilizes two antagonistic components to precisely define the boundaries of the operational window: a C-A mismatch-mediated high-pH OFF module (referred to as the Acid regulator or High pH block) which ensures the system remains in the OFF state under basic conditions, and an i-motif-mediated low-pH OFF module (referred to as the Base regulator or Low pH block) which suppresses signal when the environment becomes excessively acidic. Under acidic conditions, the self-folding of i-motif, driven by the cooperative protonation of cytosines to form intercalated hemi-protonated C-C^+^ base pairs 38,39, triggers the dissociation of the triplex complex (Acid-block: OFF). In contrast, under neutral or basic conditions, instability of the C-A mismatch limits hybridization between the core strands (Base-block: OFF). This behavior is associated with deprotonation of the A^+^-C wobble pair, which disrupts the mismatch structure that is otherwise stabilized under acidic conditions 40. By adjusting the thermodynamic balance of these two regulatory elements, stable hybridization occurs only within a narrow pH range, thereby reducing unwanted off-target responses (Figure 1B).

The modularity of the PRISM platform allows it to serve as a universal toolkit for diverse tasks (Figure 1C, i-iii). We first validated the band-pass logic and tunability of PRISM through systematic characterization, demonstrating its ability to generate non-monotonic signal responses. As a proof of concept for precision diagnostics, PRISM was applied to image subtle pH gradients in the extracellular microenvironment HeLa cells (Figure 1Ci), effectively discriminating the tumor-like acidic niche from both neutral and highly acidic backgrounds. More importantly, we showed the unique power of PRISM in achieving precise regulation of allosteric DNA Lettuce fluorogenic aptamers 41, 42 (Figure 1Cii) and logic-gated control over the CRISPR/Cas13a activity 43, 44 (Figure 1Ciii). By shifting the paradigm from monotonic switching to precision windowed control, the PRISM strategy offers a robust and scalable approach that serves as a promising foundation for exploring intelligent theranostic frameworks capable of high-fidelity disease mapping and precision molecular actuation within subtle chemical features of complex biological systems.

## Materials and Methods

**Materials and Reagents:** DNA and RNA were designed using NUPACK and purchased from Sangon Biotech Co. (Shanghai, China). The sequences are presented in Table S1. DNA oligonucleotides were purified via high-performance liquid chromatography (HPLC). HEPES, MgCl_2_, NaOAc, Tris-HCl and NaCl were purchased from Sangon Biotech Co. (Shanghai, China). WST-1 was purchased from Takara Bio USA (San Jose, CA, USA). HeLa cells were obtained from Shanghai Institutes for Biological Sciences (Chinese Academy of Science). LbuCas13a protein was purchased from Bio-lifesci (Guangzhou, China). DFHBI-1T was obtained from MedChemExpress and prepared as 1 mM DMSO stocks, stored at -20 °C for experiments.

**Instrumentations:** Fluorescence spectra and confocal images were acquired using a Rotor-Gene 6000 (Corbett Research, Australia) and a Leica TCS SP8 laser confocal microscope, respectively. Flow cytometry data were further analyzed on a Beckman Coulter CytoFLEX platform. Fluorescence scans of DNA Lettuce were performed on a Cary Eclipse Fluorescence spectrometer (Agilent) set to excite and measure emission at wavelengths of interest. DFHBI-1T was excited at 465 nm and emission measured at 480-560 nm.

**Buffer Conditions and Assembly Procedure:** All DNA oligonucleotides were redissolved in ddH_2_O. All experiments were performed in a Phy reaction buffer containing 10 mM Tris-HCl, 10 mM NaOAc, 10 mM HEPES, 100 mM NaCl, and 5 mM MgCl_2_ to mimic physiological ionic condition. The pH of the buffer is adjusted by adding HCl (1 M) and NaOH (1 M). PRISM complexes were assembled by mixing the corresponding single strands in Phy buffer at 37 °C. The DNA probes were annealed in a polymerase chain reaction (PCR) thermal cycler. The temperature was set at 95 °C for 5 min and cooled down to 20 °C by 0.1 °C/s. crRNA and blocker were annealed in Phy buffer at 85 °C for 3 min, followed by gradual cooling to 10 °C by 0.1 °C/s followed by cooling to 4 °C.

**Time-Based Fluorescence Acquisition:** Complexes were prepared in a total reaction volume of 20 μL. All fluorescence signals were subsequently monitored in the orange channel (585 nm/605 nm) using the default gain setting. All measurements were performed at 37 °C. For the PRISM logic validation, the concentration of the fluorophore-labeled strand and two or three-stranded complexes were 100 nM. Nonlinear curve fitting of the pH-dependent fluorescence profiles was performed using the asymmetric BiGaussian function in Origin 2024 software to determine the central response pH (pHc) and full width at half maximum (FWHM). The signal-to-background ratios (S/B) were calculated using the equations: S/B_1_ = F_peak_/F_pH 5.8_ and S/B_2_ = F_peak_/F_pH 7.4_. For DNA Lettuce experiments, DFHBI-1T was 1 μM and fluorescent aptamer was 500 nM. For CRISPR/Cas13a system, the reported probe concentration was 250 nM.

**Cell Culture and Cell-Surface Imaging:** HeLa cells were maintained at 37 °C in a 5% CO_2_ atmosphere using DMEM supplemented with 10% fetal bovine serum (WISENT, Cat: 086-150, Australian), 100 units of penicillin, and 100 µg/mL of streptomycin. In flow cytometry (FCM) experiment, HeLa cells (5 ×10^5^) were washed with Phy buffers at various pH values and incubated with 300 nM of the differently designed PRISM probes for 30 min at room temperature. After removing unbound probes through three sequential washes, the cells were collected by centrifugation, resuspended, and analyzed. In confocal laser scanning microscopy (CLSM) imaging, HeLa cells were seeded into 15 mm glass-bottomed dishes (1 × 10^5^ cells/dish) and cultured for 24 h. Then, the medium was replaced with 200 μL different pH buffer containing the 300 nM PRISM probe. Following a 30-min incubation, the cells were washed three times, and were visualized under the red channel (643 nm/667 nm). To investigate the incubation time, the culture medium was replaced with 200 µL of Phy buffer (pH 6.4) containing 300 nM of c-MET-anchored PRISM probe for varying time intervals (10, 20, 30, 60, and 120 min). At each indicated time point, the cells were immediately washed three times, and subsequently replenished with fresh buffer for imaging.

**WST-1 cell proliferation assay:** Cells were seeded into 96-well plates (3000 cells/well) and treated with the Premixed WST-1 Reagent (Takara Bio USA, San Jose, CA) at designated time intervals. Following a 2 h incubation period at 37 °C, the absorbance at 450 nm was recorded utilizing a BioTek EON microplate reader (USA).

### AI Assistance Disclosure

In accordance with COPE and TITAN 2025 guidelines, the Gemini large language model (Google LLC) was employed during manuscript preparation solely for text editing and grammatical refinement to enhance linguistic clarity. The tool was not utilized for data collection, simulation or figure generation, and the authors maintain full responsibility for the integrity and accuracy of the final content.

## Results and Discussion

### Rational Design of the Topological PRISM Framework

**Figure 2:**
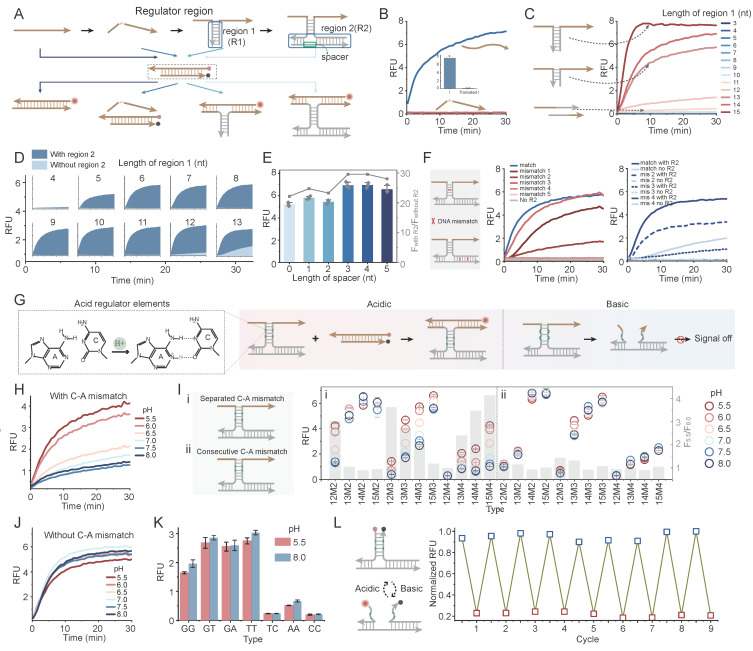
Engineering and characterization of the DNA framework and the acid-responsive PRISM gate. (A) Schematic of the DNA framework design featuring a structural nick in the single strand to control fluorescence signaling. (B) The fluorescence curve showing that the nick prevents signal output. (C) Fluorescence response as a function of the length of region 1 extension. Region 2 is 26nt. (D) Comparison of signal output with or without region 2 across various region 1 lengths. (E) Optimization of the spacer length between region 1 and region 2. (F) Effect of single-base mismatches in region 2 (left) and region 1(right) on the overall hybridization efficiency. Region 1 is 13nt, region 2 is 26nt. (G) Molecular mechanism of the acid-gate: introduction of pH-sensitive C-A mismatches into region 1. (H) Fluorescence kinetics of the acid-gate (12-nt region 1, 26-nt region 2, 2 C-A mismatches) across a pH range of 5.5 to 8.0. (I) Programmability of the Acid-gate: Effect of C-A mismatch arrangement and quantity on the pH-dependent signal profile (colored circles). (J) Control experiment using a 13-nt region 1 without C-A mismatches. (K) Specificity test across diverse mismatch types. (L) Reversibility and reset performance over multiple cycles between pH 8.0 (blue) and pH 5.5 (red). Data represent mean ± S.D.; n = 3.

To achieve high-fidelity band-pass responsiveness, we first engineered a programmable DNA hybridization framework based on the toehold-mediated strand displacement (TMSD) (Figure 2A). In this design, the presence of a structural nick within the DNA strand prevents the dissociation of fluorophore and quencher, resulting in a OFF state (Figure 2B). Signal generation is initiated by extending the sequence across the nick to form the first regulatory domain (region 1). We observed that the hybridization stability was highly dependent on the length of this domain. A short region 1 (e.g., < 12 nt) resulted in negligible fluorescence output (Figure 2C). Mismatches in region 1 can also significantly disrupt hybridization (Figure S1). To enhance the thermodynamic stability of the complex, a second regulatory domain (region 2) was introduced. The synergistic interaction between these two regions revealed that the shorter the region 1, the more the system relied on the presence of region 2 to maintain stable hybridization and generate a detectable signal (Figure 2D). The contribution of region 2 to the stability of the three-stranded complex structure has also been demonstrated in different lengths of region 2 (Figure S2-S4). The flexible spacer between the two regions also played an important role. These flexible linkers have alternating A/T composition within 1-5 nt, and 3-4 nt variants yield the sufficient conformational freedom (Figure 2E, Figure S5). This design minimizes thermodynamic affinity, effectively preventing non-specific hybridization with the core strands. Furthermore, site sensitivity was confirmed by introducing mismatches into either region 1 or region 2 (Figure 2F). When there is mismatch on both the left and right sides of spacer in region 2, the fluorescence signal was significantly reduced. When there are three or more mismatches in region 1, the fluorescence signal was also significantly reduced, highlighting the precision of our modular assembly.

### Engineering the Acid-Responsive Logic via Programmable C-A Mismatches

Based on the optimized framework, the high-pH block (Acid regulator) was constructed by introducing pH-sensitive C-A mismatches into region 1 (Figure 2G). Under acidic conditions, the triplex complex was stabilized by the A^+^-C wobble pair and fluorescence is activated (ON state). Under neutral or basic conditions, reduced stability of non-protonated C-A mismatches promotes dissociation of region 1, destabilizing the overall assembly and suppressing signal generation (OFF state). Kinetic studies of a representative configuration containing a 12 nt region 1 with two C-A mismatches and a 26 nt region 2 showed an acid-on response. As the pH decreased from 8.0 to 5.5, fluorescence gradually saturated (Figure 2H).

To examine the tunability of the acid-gate, both the arrangement (consecutive or separated) and number of C-A mismatches in region 1 were varied (Figure 2I, Figure S6-S9). Signal responses across a pH gradient, quantified as F_5.5_/F_8.0_ (grey bars), indicated that the activation threshold shifted with sequence design. Among the tested configurations, 12M2 (12-nt region 1 containing two C-A mismatches) showed stronger pH responsiveness than longer variants (13M2-15M2). This difference likely reflects the higher stability of hybridization domains, which reduces switching efficiency under pH regulation. In contrast, the responsiveness was reduced by excessive mismatch density in 12-nt domains (12M3 and 12M4), as the scarcity of complementary bases led to structural instability even under acidic conditions. Although the F_5.5_/F_8.0_ of 12M3 is high, the overall signal is low. Considering the acidic response effect and the absolute fluorescence signal, the optimal mismatch and length pair were proposed: 2 C-A pairs for 12 nt, 3 C-A pairs for 13-14 nt, and 4 C-A pairs for 15 nt. Furthermore, separated C-A arrangements generally demonstrated enhanced pH sensitivity over consecutive ones, because consecutive mismatches may impose a more severe thermodynamic penalty on the hybridized complex.

A control group containing a 13 nt region 1 without C-A mismatches showed no detectable pH-dependent signal variation, indicating that the framework does not respond to pH changes (Figure 2J). The pH-responsive behavior was removed through replacing C-A mismatch with other mismatch types (e.g., G-G, G-T), suggesting a specific contribution of C-A mismatch to the observed response (Figure 2K). Then, the system demonstrated exceptional reversibility by alternating a turn-OFF assay, with signal quenching corresponding to complex assembly at pH 5.5 and recovery matching dissociation at pH 8.0 (Figure 2L). The pH-responsive assembly was also confirmed by native polyacrylamide gel electrophoresis (PAGE), shifting from a prominent hybridized complex at pH 5.5 to a partial dissociation equilibrium at pH 6.4, and finally to complete disassembly at pH 7.4 (Figure S10). This provided structural evidence for the pH-dependent deprotonation and disruption of the A^+^C wobble pairs.

### Development of the Base Regulator via pH-Dependent i-Motif Folding

**Figure 3:**
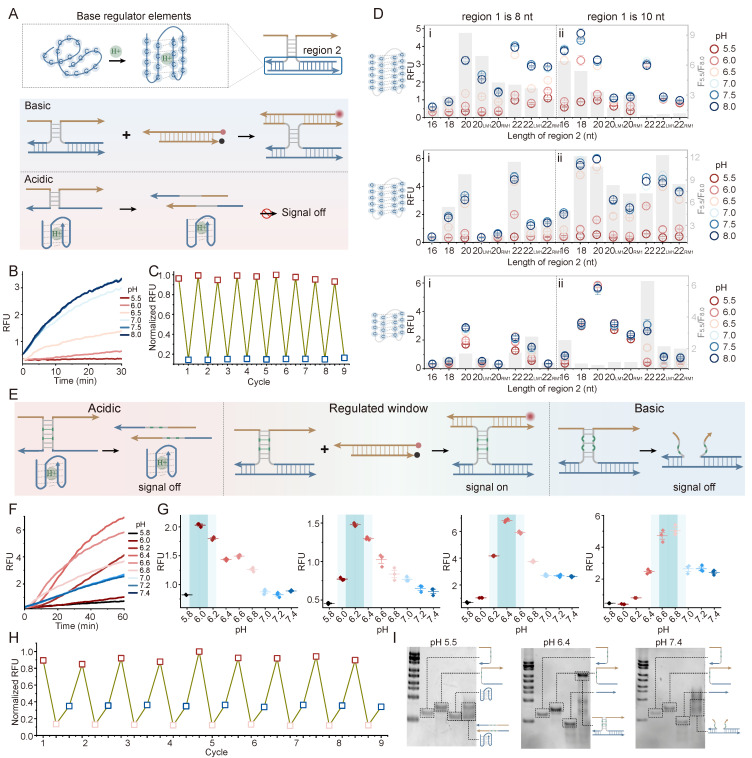
Engineering and characterization of the base regulator and the integrated PRISM band-pass filter. (A) Mechanism of the Base regulator (low-pH block). i-motif folding leads to strand dissociation and signal silencing. (B) pH-dependent fluorescence kinetics for a representative C6 i-motif system. (C) Reversibility of the base-regulator in a quenching-based assay (Assembled: pH 8.0, blue; Dissociated: pH 5.5, red). (D) Systematic experiment of i-motif types (C6, C5, C4) and framework topology (region 1: 8 vs 10 nt; region 2: 16–22 nt). Grey bars represent the switching ratio (F_8.0_/F_5.5_). LM1 refers to a mismatch on the left side of spacer in region 2, and RM1 indicates a mismatch on the right side of the spacer. (E) Conceptual overview of the integrated PRISM strategy. Synergistic Acid and Base regulators define a narrow operational window. (F) Band-pass fluorescence kinetics of the optimized PRISM probe. (G) Programmability of PRISM with different i-motif type and region 1. (H) Multi-state reversibility testing across target (pH 6.4, pink) and non-target (pH 5.5, pH 8.0, red and blue) environments using a turn-off sensor design. (I) PAGE validation of pH-selective complex formation. Data represent mean ± S.D.; n = 3.

To introduce a complementary base-regulated module, a low-pH block (Base regulator) was constructed by incorporating pH-sensitive i-motif into region 2 (Figure 3A). Under neutral or basic conditions, the i-motif sequence remains in a random-coil state, permitting stable three-strand hybridization and signal generation. Under acidic conditions, the sequence folds into a C:C^+^ intercalated i-motif structure, promoting dissociation of the regulatory strand (Figure 3A). This high-pass (base-on) pH responsiveness was confirmed by a prototype system, featuring a 6-cytosine cluster (C6) i-motif and an 8 nt region 1(Figure 3B). The exceptional reversibility of this base-regulator was validated via a turn-OFF assay, where signal quenching coincided with complex assembly at pH 8.0 and recovery followed dissociation at pH 5.5 (Figure 3C).

Systematic characterization revealed that the switching performance (quantified by the F_8.0_/F_5.5_, grey bars) is governed by a thermodynamic balance between the hybridization energy of the framework and the folding force of the i-motif (Figure 3D, Figure S11-S13). Higher-order i-motif clusters (C6 and C5) outperformed the C4 variant, as the increased number of cytosine provides a stronger breaking force. Folding energy of C4 variant was insufficient to overcome the hybridization of three-strand assembly. Different i-motif clusters showed distinct sensitivity profiles when the region 1 was extended to 10 nt. For the C6 variant, which possesses the highest folding stability, a decrease in switching performance was observed as region 2 was lengthened. For the C6 design, an overly stable framework appeared to reduce efficient pH-triggered dissociation, likely because of increased thermodynamic constraints. In contrast, the C5 variant maintained relatively high switching ratios even when the length of region 2 was increased. These results indicate that base regulator performance does not depend on a single sequence design but can arise from appropriate energy matching between the regulatory domain and DNA scaffold. The i-motif-mediated low-pH OFF behavior was also supported through PAGE analysis. At pH 5.5, i-motif folding was associated with substantial disassembly, whereas partial hybridization was observed at pH 6.4 and a clear three-strand complex appeared at pH 7.4 (Figure S14).

### Implementation and Tunability of the PRISM Molecular Band-Pass Filter

The band-pass filter was assembled by combining the acid regulator and the base regulator into a single framework (Figure 3E). At low pH, folding of the i-motif promotes strand dissociation, whereas C-A mismatches destabilize the complex under neutral or alkaline conditions. Stable hybridization and fluorescence output occur only within the pH range where neither regulatory block is activated. A band-pass response with peak center at pH 6.4 was showed using a representative PRISM configuration containing C6 i-motif and 12M2 region 1 (Figure 3F). Although equilibration required 30-60 min and may limit detection of rapid pH fluctuations, the response remained compatible with relatively stable tumor microenvironments maintained by metabolic homeostasis. Kinetics could be improved by adjusting toehold lengths or using loop-linear strand displacement. The response window of PRISM could also be altered through sequence modification. Changes in either region 1 or region 2 could shift the fluorescence profile, indicating tunable system behavior (Figure 3G, Figure S15).

Then, pH-dependent fluorescence curves were analyzed using an asymmetric BiGaussian fitting model. Parameters included central response pH (pHc), full width at half maximum (FWHM), acidic signal-to-background ratio (S/B_1_, ratio of peak fluorescence to fluorescence at pH 5.8), and physiological signal-to-background ratio (S/B_2_, ratio of peak fluorescence to fluorescence at pH 7.4) (Figure S15). The fitted results showed that pHc shifted with sequence variation, changing from pH 6.0 (PRISM 1) to 6.2 (PRISM 2), 6.4 (PRISM 3 and 4), 6.5 (PRISM 5), and reaching 6.8 (PRISM 6). Window offsets (ΔpH up to +0.3 units) was produced by sequence change, enabling pH targeting across the sub-acidic range. Expanding the responsive core from a C4 in PRISM 1 to C6 in PRISM 2-6 strengthened internal base stacking within the i-motif structure. Correspondingly, the acidic signal-to-background ratio increased from 2.47-fold to 13.31-fold.

While PRISM 2-6 share identical C-core lengths, their differences in pHc positions and signal contrast indicated that flanking duplex and C-A mismatches contribute to changing the free-energy landscape. For instance, PRISM 3 and PRISM 4 displayed the same pHc value at 6.4, whereas conformational flexibility was reduced by the compaction of flanking domains in PRISM 4, decreasing the FWHM from 1.04 to 0.80. In addition, hybridization stability was improved through reducing the number of C-A mismatches from three of PRISM 2 to two of PRISM 3, shifting pHc toward a less acidic regime and increasing the turn-on contrast (S/B_1_). These results indicated that adjustment of DNA motifs enables predictable and narrow-band pH response.

Next, the multi-state reversibility of the PRISM filter was evaluated (Figure 3H). Using the quenching-based assay, the system repeatedly switched between the Window-ON state (pH 6.4, bottom pink dots) and OFF states (pH 8.0 and pH 5.5, upper blue and red dots). Moreover, PRISM assembly was examined using PAGE (Figure 3I). At pH 5.5 and 7.4, no clear bands corresponding to the three-strand complex were observed. By contrast, a high-molecular-weight band appeared at pH 6.4, indicating that assembly occurred only within the programmed pH window. These findings support the ability of PRISM to selectively operate across chemically heterogeneous environments.

### Programmable pH Mapping on Cell Surfaces via PRISM Modularity

**Figure 4:**
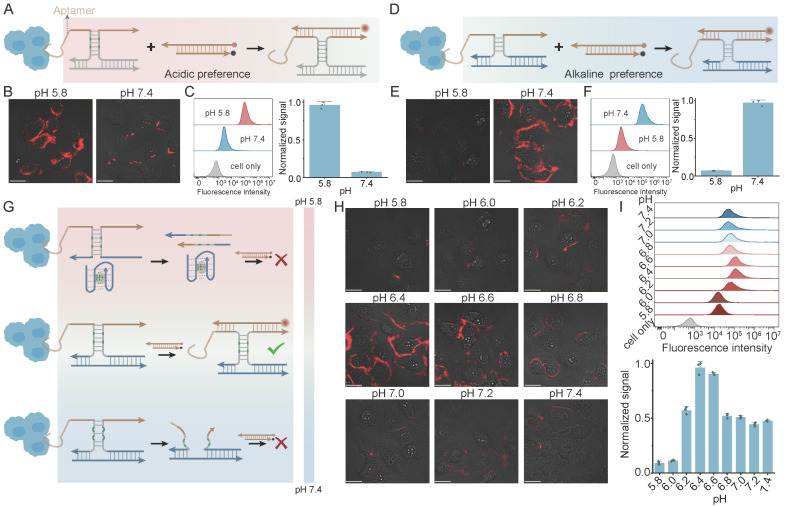
Spatially regulated extracellular pH sensing on HeLa cells via aptamer-anchored PRISM logic. (A) Schematic of the aptamer-anchored acidic-preference module utilizing pH-sensitive C-A mismatches for acid-ON signaling. (B, C) CLSM images (B) and flow cytometry (FCM) analysis (C) on HeLa cell membranes at pH 5.8 and pH 7.4 (D) Mechanism of the aptamer-anchored base-preference module based on i-motif folding. (E, F) CLSM images (E) and FCM characterization (F) verifying base-ON response logic on the HeLa cell interface. (G) Conceptual design of the integrated PRISM narrow-window module, combining antagonistic regulators for band-pass logic on the cell surface. (H, I) pH-dependent confocal imaging (H) and multi-point FCM (I) on HeLa cells. Scale bars: 20 μm. Data represent mean ± S.D.; n = 3.

The modularity of DNA nanotechnology allows the PRISM platform to be seamlessly integrated with cell-targeting motifs for spatially regulated imaging. The c-MET-binding aptamer was utilized as a specific anchor to tether PRISM probe onto the surface of HeLa cells. We first examined the individual regulatory modules on the HeLa cell surface. The acidic-preference module based on pH-sensitive C-A mismatches, displayed an acid-on response pattern (Figure 4A). CLSM showed clear membrane-localized fluorescence at pH 5.8, while fluorescence was reduced under neutral physiological conditions (pH 7.4) (Figure 4B). FCM analysis also showed a rightward shift of the fluorescence population at pH 5.8 (Figure 4C). In contrast, an opposite response pattern was observed through the i-motif-based base regulator (Figure 4D). At pH 7.4, the i-motif remained in a random-coil state, allowing probe assembly and fluorescence generation on the membrane. Under acidic conditions, dissociation of the probe components was driven by self-folding of the i-motif (Figure 4E). FCM data further supported this base-on behavior, with approximately tenfold fluorescence difference between the two states (Figure 4F).

By synergistically integrating the i-motif and C-A mismatch logic within the aptamer-anchored framework, the system defined an exclusive operational window centered at pH 6.4, a value relevant to the tumor extracellular (Figure 4G). A non-monotonic signal pattern was demonstrated in CLSM imaging: fluorescence was minimal at both the acidic extreme (pH 5.8) and the neutral baseline (pH 7.4), but reached a maximum within the regulated window (Figure 4H). This band-pass behavior was also validated through FCM across a gradient pH, with the highest signal density observed only at pH 6.4 (Figure 4I). Then, the incubation time of the PRISM probe on HeLa cell surfaces were evaluated (10, 20, 30, 60, and 120 min) at pH 6.4 via CLSM (Figure S16). Within the early 30 min window, the fluorescence remained strictly confined to the cell periphery. Prolonged incubation (60-120 min) led to a progressive decline in membrane fluorescence, presumably because receptor-mediated endocytosis shuttles the probes into highly acidic lysosomal compartments, thereby leading the i-motif-mediated low-pH OFF module. Thus, a 30-min incubation period was chosen to ensure the high fidelity of extracellular pH sensing.

The unmodified aptamer is entirely pH-insensitive, resulting in non-selective fluorescence regardless of the microenvironmental pH (Figure S17A). To verify the anchoring specificity, a random sequence of aptamer was evaluated under identical conditions, which resulted in a complete absence of cell-surface fluorescence (Figure S17B). Notably, these cell-surface experiments were conducted under controlled pH buffer gradients to verify operational boundaries of the PRISM probe. This well-defined model system establishes a necessary baseline prior to navigating unbuffered, endogenously acidified microenvironments. Moreover, the negligible cytotoxicity was confirmed through WST-1 assays (Figure S18). These results indicated that PRISM strategy provides a generalizable tool for the discriminatory sensing of subtle chemical signatures on cancer cell surfaces.

### Allosteric Actuation of Fluorogenic Aptamers via PRISM Logic

**Figure 5:**
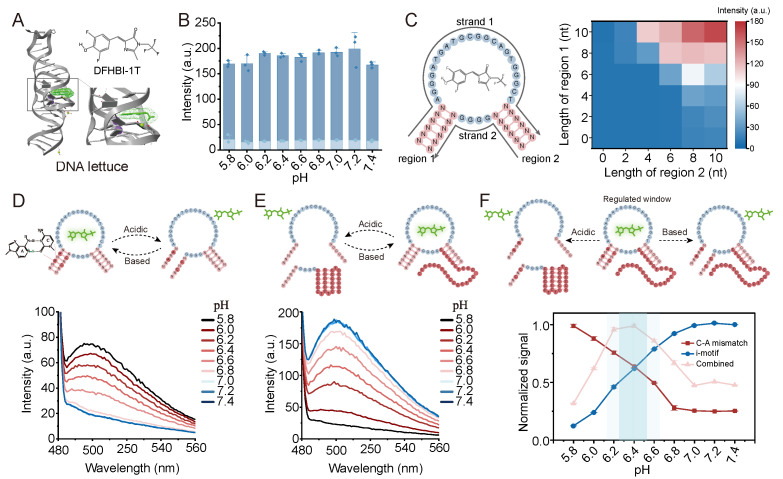
Allosteric control of fluorogenic DNA Lettuce aptamer via PRISM logic. (A) Schematic of the DNA Lettuce aptamer and its fluorogenic substrate DFHBI-1T. (B) pH-independence of the intact DNA Lettuce and free DFHBI-1T dye across physiological ranges. (C) Design of the split DNA Lettuce system. The arrow is from DNA terminal 5 to terminal 3. The heatmap shows the orthogonal optimization of hybridization lengths for region 1 and region 2. (D) Implementation of the Acidic-ON actuator through incorporation of C-A mismatches in region 1. (E) Implementation of the Base-ON actuator with i-motif in region 2. (F) Realization of the Band-pass actuator. Data represent mean ± S.D.; n = 2.

To further demonstrate the versatility of PRISM, it was applied to the allosteric regulation of DNA Lettuce, a fluorogenic aptamer that exhibits high brightness upon binding DFHBI-1T substrate (Figure 5A). We first conducted control experiments to ensure that the intact DNA Lettuce and the dye are pH-independent (Figure 5B, Figure S19). Drawing inspiration from the rational design of allosteric DNA nanostructures, the split fluorogenic aptamer was developed, where the core Lettuce sequence is divided into two non-functional fragments (Figure 5C). The restoration of fluorescence is dependent on the simultaneous hybridization of two regulatory domains, which correspond to region 1 and region 2 of the PRISM framework. A heatmap changing the lengths of two regions showed cooperative effects between them. Detectable fluorescence was observed only when one of the two regions reached a minimum length of 8 nt, indicating a thermodynamic requirement for signal activation (Figure 5C, Figure S20).

Based on this split system design, PRISM regulatory elements were incorporated into the DNA Lettuce framework. Introduction of C-A mismatches into region 1 produced an acidic-ON actuator, which formed the active Lettuce structure and generated fluorescence under acidic conditions (Figure 5D). In contrast, a base-ON actuator was generated by insertion of the i-motif cluster into region 2. The folding of the i-motif at low pH prevents aptamer assembly, while its random-coil state at high pH allows fluorescence recovery (Figure 5E). Then, the complete PRISM strategy was implemented by combining both modules. Fluorescence curves demonstrated that the DNA Lettuce could be precisely switched on within the pre-programmed narrow pH window, while remaining silenced at both extreme acidic and neutral pH conditions (Figure 5F, Figure S21). These results confirmed that PRISM can serve as a robust biological software to allosterically control the function of complex DNA-based devices.

### Logic-Gated Control of CRISPR/Cas13a Activity via PRISM Integration

**Figure 6:**
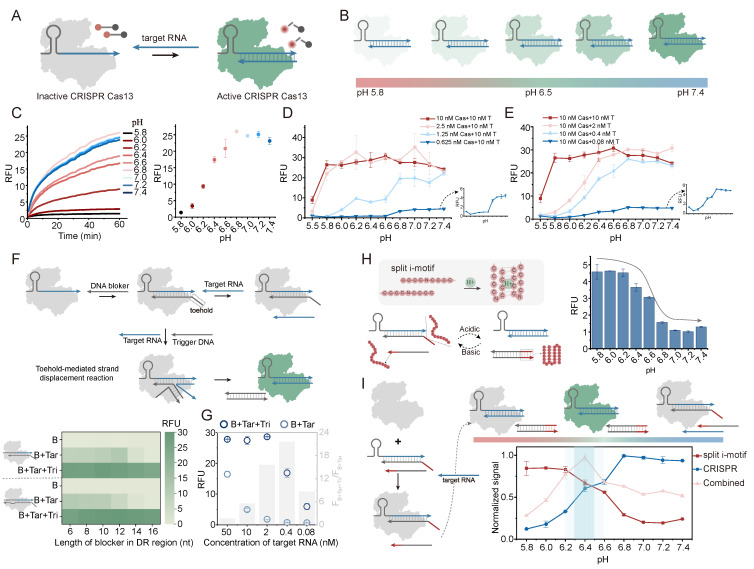
Precision control of CRISPR/Cas13a activity via integrated PRISM logic. (A) Schematic of LbuCas13a activation. Target RNA binding triggers trans-cleavage of RNA reporters. (B, C) Intrinsic basic-preference of LbuCas13a: (B) conceptual illustration and (C) pH-dependent fluorescence kinetics and endpoint signals. (D, E) Effect of Cas (D) and target RNA (E) concentrations on the intrinsic pH response. (F) TMSD-based control of crRNA. Top: mechanism of blocking and trigger-induced deblocking. Heatmap: optimization of blocking length and position (3'-end vs. 5'-end of DR) to balance leakage (B+Tar) and activation (B+Tar+Tri). The spacer region of crRNA is 20nt. (G) Optimization of target RNA concentration using the 14-nt 5'-DR blocker. (H) Engineering the Acidic-ON actuator through introduction of a split i-motif into the toehold region. (I) Implementation of the band-pass CRISPR actuator: synergistic dual-suppression at extreme pH values confines CRISPR activity within a narrow window. Data represent mean ± S.D.; n = 2.

A key objective of the PRISM strategy is to couple pH-responsive regulation with biological systems. The regulation of LbuCas13a was experimented, a programmable RNA-guided ribonuclease known for trans-cleavage activity following target RNA recognition (Figure 6A). The intrinsic pH sensitivity of LbuCas13a system was characterized. The enzyme displayed a natural base preference with a decreased fluorescence signal as pH shifted from basic to acidic conditions (Figure 6B, 6C). This pH-dependent activity remained consistent across various concentrations of Cas enzyme and target RNA (Figure 6D, 6E and Figure S22), indicating that Cas13a may serve as a natural base regulator in our PRISM framework.

To generate an acid-responsive module, TMSD strategy was utilized to control the accessibility of the crRNA, based on conditional guide RNA design. By designing a DNA blocking strand that hybridizes with the direct repeat (DR) region of the crRNA, the binding of the target RNA is sterically hindered, maintaining the Cas enzyme in an inactive state (Figure 6F). Upon the introduction of a trigger DNA, the blocker is removed via TMSD, allowing Cas activation. To optimize the blocking effect and triggering kinetics, different blocking positions and lengths were tested. We observed that the background signal (B+Tar) was inversely proportional to the length of blocker, as longer sequences provided higher thermodynamic barriers. The spacer lengths of crRNA were also compared, and the blocking effect of 20 nt spacer was better than that of 28 nt (Figure S23-25). The 14-nt blocker targeting the 5'-end of DR region balanced low leakage with efficient activation (Figure 6F). The 0.4 nM target RNA was chosen as the optimal concentration to maintain a high signal-to-background ratio (Figure 6G, Figure S26).

An acidic-ON CRISPR actuator was generated by introducing a split i-motif into the toehold region of the TMSD reaction (Figure 6H). The split i-motif only assembles and stabilizes the toehold binding under acidic conditions, thereby enabling strand displacement and fluorescence output (Figure S27a). An obvious acid-responsive activation profile was observed through fluorescence measurements (Figure 6H, right). Finally, by combining the split i-motif-driven "acidic-ON" logic with the intrinsic "base-ON" activity of Cas enzyme, a band-pass CRISPR actuator was obtained (Figure 6I). In excessively acidic environments, although the TMSD reaction was triggered by i-motif folding, the intrinsic enzymatic activity of Cas13a was suppressed. Under basic conditions, the TMSD reaction is inhibited due to the instability of split i-motif toehold. CRISPR activity is confined to a narrow pH window, where engineered DNA regulation overlaps with the intrinsic pH preference of the protein (Figure 6I, Figure S27b). This strategy which combines synthetic DNA control with intrinsic protein sensitivity, may support the development of environmentally responsive bio-integrated systems.

## Conclusion

In summary, we have developed PRISM (pH-Responsive DNA Inter-Strand Matching) as a multifunctional and programmable DNA platform for executing precision actuation within narrow pH windows. By integrating the antagonistic thermodynamic properties of i-motif-based low-pH blocks and C-A mismatch-based high-pH blocks into a three-strand DNA framework, we demonstrated an effective strategy to convert biological pH signals into non-monotonic molecular responses. This is the first report to demonstrate a method for engineering programmable narrow-window molecular actuation within a purely DNA-based framework. Unlike conventional monotonic off-to-on DNA switches, PRISM operates as a molecular band-pass filter that restricts functional activity to defined pH windows and may reduce unintended activation in neutral healthy tissues as well as highly acidic intracellular compartments.

The modular nature of DNA architecture enabled the application of PRISM logic to different biological functions, from diagnostic imaging to the regulation of enzymatic activity. Its utility was demonstrated in the mapping of subtle pH gradients on HeLa cell surfaces and the allosteric regulation of the fluorogenic DNA Lettuce aptamer. Importantly, by interfacing synthetic DNA logic with the intrinsic pH sensitivity of LbuCas13a, a narrow window regulation of CRISPR enzymatic activity was achieved. This integration highlights the unique advantage of DNA systems in providing a native language for communicating with complex biological machineries without requiring complex and potentially immunogenic synthetic bioconjugations.

The PRISM strategy offers a scalable tool that may conceptually inspire the design of next-generation intelligent, bio-integrated theranostic drugs. Given its sequence-level programmability and inherent biocompatibility, PRISM serves as a robust conceptual framework for exploring adaptive diagnostic-to-actuation models, while offering the potential to be sequence-adapted for room-temperature operations *in vitro* diagnostics. To facilitate future *in vivo* applications, the biostability of the PRISM framework against nuclease degradation can be further enhanced through established chemical modifications, such as phosphorothioate linkages or locked nucleic acids. We anticipate that this windowed control paradigm holds great promise for advancing precision diagnostics and spatially regulated molecular actuation, enabling the execution of complex biological tasks with environmental fidelity in subtle pathological landscapes.

## Supplementary Material

Supplementary figures and tables.

## Data Availability

The data generated in this study are available upon request from the corresponding author.

## Abbreviations

CLSM: confocal laser scanning microscopy
DR: direct repeat
FCM: flow cytometry
FWHM: full width at half maximum
PRISM: pH-Responsive DNA Inter-Strand Matching
S/B: signal-to-background ratio
TMSD: toehold-mediated strand displacement

## References

[1] Purvis JE, Lahav G (2013). Encoding and decoding cellular information through signaling dynamics. Cell.

[2] Wang C, Yue L, Willner I (2020). Controlling biocatalytic cascades with enzyme-DNA dynamic networks. Nat Catal.

[3] Fan P, Liu Y, Pan Y, Ying Y, Ping J (2025). Three-dimensional micro- and nanomanufacturing techniques for high-fidelity wearable bioelectronics. Nat Rev Electr Eng.

[4] Buller R, Lutz S, Kazlauskas RJ, Snajdrova R, Moore JC, Bornscheuer UT (2023). From nature to industry: Harnessing enzymes for biocatalysis. Science.

[5] Bell E L, Finnigan W, France S P, Green A P, Hayes M A, Hepworth L J (2021). Biocatalysis. Nat Rev Methods Primers.

[6] Manco Urbina PA, Paradisi A, Hasler R, Sensi M, Berto M, Saygin GD (2024). Dynamic studies of antibody-antigen interactions with an electrolyte-gated organic transistor. Cell Rep Phys Sci.

[7] Burkhardt DB, Stanley JS 3rd, Tong A, Perdigoto AL, Gigante SA, Herold KC (2021). Quantifying the effect of experimental perturbations at single-cell resolution. Nat Biotechnol.

[8] Wang Y, Jiang Z, Kwon SH, Ibrahim M, Dang A, Dong L (2026). Flexible Sensor-Based Human-Machine Interfaces with AI Integration for Medical Robotics. AdAdv Robot Res.

[9] Tapia-Rojo R, Alonso-Caballero Á, Fernández JM (2020). Talin folding as the tuning fork of cellular mechanotransduction. Proc Natl Acad Sci U S A.

[10] Webb BA, Chimenti M, Jacobson MP, Barber DL (2011). Dysregulated pH: a perfect storm for cancer progression. Nat Rev Cancer.

[11] Hamm LL, Nakhoul N, Hering-Smith KS (2015). Acid-Base Homeostasis. Clin J Am Soc Nephrol.

[12] Rodrigues RC, Ortiz C, Berenguer-Murcia Á, Torres R, Fernández-Lafuente R (2013). Modifying enzyme activity and selectivity by immobilization. Chem Soc Rev.

[13] Estrella V, Chen T, Lloyd M, Wojtkowiak J, Cornnell HH, Ibrahim-Hashim A (2013). Acidity generated by the tumor microenvironment drives local invasion. Cancer Res.

[14] Boedtkjer E, Pedersen SF (2020). The Acidic Tumor Microenvironment as a Driver of Cancer. Annu Rev Physiol.

[15] Freeman SA, Grinstein S, Orlowski J (2023). Determinants, maintenance, and function of organellar pH. Physiol Rev.

[16] Casey JR, Grinstein S, Orlowski J (2010). Sensors and regulators of intracellular pH. Nat Rev Mol Cell Biol.

[17] Steinegger A, Wolfbeis OS, Borisov SM (2020). Optical Sensing and Imaging of pH Values: Spectroscopies, Materials, and Applications. Chem Rev.

[18] Zhang Y, Takahashi Y, Hong SP, Liu F, Bednarska J, Goff PS (2019). High-resolution label-free 3D mapping of extracellular pH of single living cells. Nat Commun.

[19] Grashei M, Wodtke P, Skinner JG, Sühnel S, Setzer N, Metzler T (2023). Simultaneous magnetic resonance imaging of pH, perfusion and renal filtration using hyperpolarized (13)C-labelled Z-OMPD. Nat Commun.

[20] Düwel S, Hundshammer C, Gersch M, Feuerecker B, Steiger K, Buck A (2017). Imaging of pH in vivo using hyperpolarized (13)C-labelled zymonic acid. Nat Commun.

[21] Liu A, Huang X, He W, Xue F, Yang Y, Liu J (2021). pHmScarlet is a pH-sensitive red fluorescent protein to monitor exocytosis docking and fusion steps. Nat Commun.

[22] Wang DY, Yang G, van der Mei HC, Ren Y, Busscher HJ, Shi L (2021). Liposomes with Water as a pH-Responsive Functionality for Targeting of Acidic Tumor and Infection Sites. Angew Chem Int Ed Engl.

[23] Di Z, Lu X, Zhao J, Jaklenec A, Zhao Y, Langer R (2022). Mild Acidosis-Directed Signal Amplification in Tumor Microenvironment via Spatioselective Recruitment of DNA Amplifiers. Angew Chem Int Ed Engl.

[24] Zhang J, Wang D, Chen H, Yuan X, Jiang X, Ai L (2022). A pH-Responsive Covalent Nanoscale Device Enhancing Temporal and Force Stability for Specific Tumor Imaging. Nano Lett.

[25] Lu C, Li Z, Wu N, Lu D, Zhang X-B, Song G (2023). Tumor microenvironment-tailored nanoplatform for companion diagnostic applications of precise cancer therapy. Chem.

[26] Yang Y, Chen B, Wan F, Fu C, Chen M, Ma B (2025). Cascade-heterogated proton nanotransistors for multiplex pH-interval imaging. Nat Commun.

[27] Mao D, Dong Z, Liu X, Li W, Li H, Gu C (2024). An Intelligent DNA Nanoreactor for Easy-to-Read In Vivo Tumor Imaging and Precise Therapy. Angew Chem Int Ed Engl.

[28] Mirkin CA, Petrosko SH (2023). Inspired Beyond Nature: Three Decades of Spherical Nucleic Acids and Colloidal Crystal Engineering with DNA. ACS Nano.

[29] Zhang L, Liu Q, Guo Y, Tian L, Chen K, Bai D (2024). DNA-based molecular classifiers for the profiling of gene expression signatures. J Nanobiotechnology.

[30] He L, Zheng N, Wang Q, Du J, Wang S, Cao Z (2022). Responsive Accumulation of Nanohybrids to Boost NIR-Phototheranostics for Specific Tumor Imaging and Glutathione Depletion-Enhanced Synergistic Therapy. Adv Sci (Weinh).

[31] Yuan K, Meng H-M, Wu Y, Chen J, Xu H, Qu L (2022). Extracellular Milieu and Membrane Receptor Dual-Driven DNA Nanorobot for Accurate in Vivo Tumor Imaging. CCS Chem.

[32] Li L, Xu S, Peng X, Ji Y, Yan H, Cui C (2021). Engineering G-quadruplex aptamer to modulate its binding specificity. Natl Sci Rev.

[33] Guo Y, Yao D, Zheng B, Sun X, Zhou X, Wei B (2020). pH-Controlled Detachable DNA Circuitry and Its Application in Resettable Self-Assembly of Spherical Nucleic Acids. ACS Nano.

[34] Li T, Famulok M (2013). I-motif-programmed functionalization of DNA nanocircles. J Am Chem Soc.

[35] Han X, Dong X, Lin X, Yu H, Zhang L, Wang W (2025). Rational design of tunable pH switches through shadow-strand hybridization-actuated displacement engineering. Nucleic Acids Res.

[36] Yang D, Hartman MR, Derrien TL, Hamada S, An D, Yancey KG (2014). DNA materials: bridging nanotechnology and biotechnology. Acc Chem Res.

[37] Shen L, Wang P, Ke Y (2021). DNA Nanotechnology-Based Biosensors and Therapeutics. Adv Healthc Mater.

[38] Shi L, Peng P, Du Y, Li T (2017). Programmable i-motif DNA folding topology for a pH-switched reversible molecular sensing device. Nucleic Acids Res.

[39] Nesterova IV, Nesterov EE (2014). Rational design of highly responsive pH sensors based on DNA i-motif. J Am Chem Soc.

[40] Fu W, Tang L, Wei G, Fang L, Zeng J, Zhan R (2019). Rational Design of pH-Responsive DNA Motifs with General Sequence Compatibility. Angew Chem Int Ed Engl.

[41] Zhang T, Qian X, Zhang J, Cui H, Bai T, Wang W (2026). Light-Up Nanostructures with Allosterically Controlled Fluorogenic DNA Aptamers. Adv Mater.

[42] Zhang L, Feng T, Liu Q, Zuo C, Wu Y, Zhao H (2025). Engineering thermostable fluorescent DNA aptamer for the isothermal amplification of nucleic acids. Biosens Bioelectron.

[43] Larsen BB, Kimchi O, Dunkley ORS, Grimm MS, Siegers JY, Huang Y (2025). RNA structure modulates Cas13 activity and enables mismatch detection. Nat Biotechnol.

[44] Sinha S, Molina Vargas AM, Arantes PR, Patel A, O'Connell MR, Palermo G (2024). Unveiling the RNA-mediated allosteric activation discloses functional hotspots in CRISPR-Cas13a. Nucleic Acids Res.

