# Supplementary material for: Programmable pH-responsive DNA inter-strand matching (PRISM) for precision molecular band-pass actuation
Source: Theranostics. 2026 Jun 17;16(13):7613–25. doi: 10.7150/thno.136316 (PMC13295757; doi:10.7150/thno.136316)
Supplement: Supplementary file 1 — Supplementary figures and tables. [file thnov16p7613s1.pdf]

Supporting Information

Programmable pH-Responsive DNA Inter-Strand Matching (PRISM) for Precision Molecular Band-Pass Actuation

Xiaole Han[1,2#], Hongyan Yu[1,#], Xiaomei Lin[1,#], Li Zhang[1,#], Weitao Wang[1], Yaoyi Zhang[1], Jianbo Jiang[1], Xingyu Liu[1] , Ke Lv[*,1] and Guoming Xie[*,1,2]

[1]Department of Neurosurgery, Laboratory of Neurological Diseases and Interdisciplinary Medicine, The First Affiliated Hospital of Chongqing Medical University, No.1 Youyi Road, Chongqing, 400016, China.

[2]Key Laboratory of Clinical Laboratory Diagnostics (Chinese Ministry of Education), College of Laboratory Medicine, Chongqing Medical University, Chongqing, 400016, PR China.

* To whom correspondence should be addressed. Tel: +86 23 68485240; Fax: +86 23 68485239; Email: guomingxie@cqmu.edu.cn. Correspondence may also be addressed to Ke Lv Email: luke@hospital.cqmu.edu.cn.

[#]The authors wish it to be known that, in their opinion, the authors should be regarded as Joint First Authors.

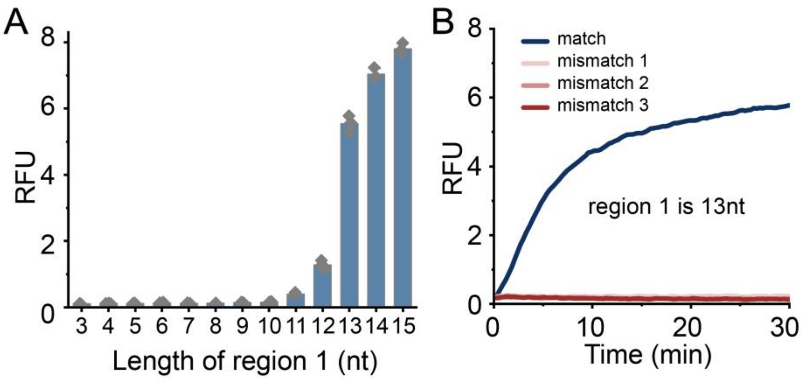

Figure S1. Fluorescence of region 1 of different lengths (A) and the effect of introducing mismatch in region 1 (B).

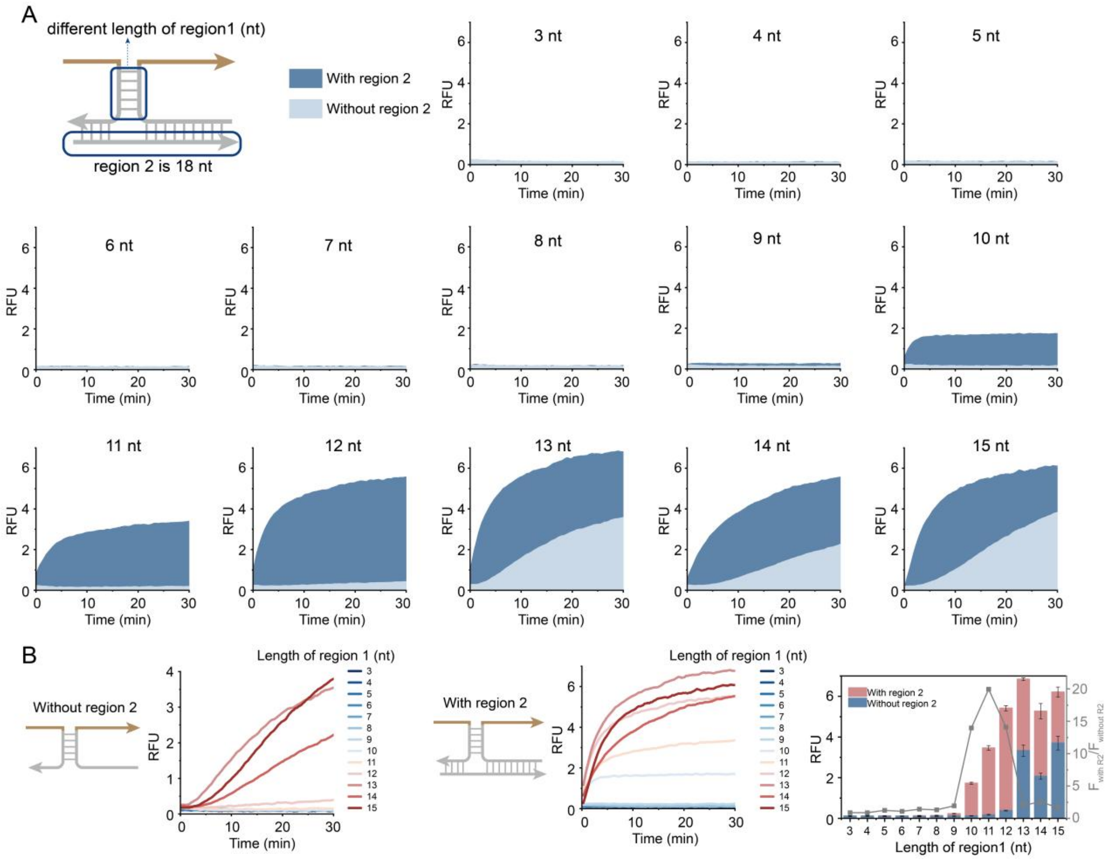

Figure S2. When region 2 is 18nt, the fluorescence curves of region 1 of different lengths (A), and the signal comparison of whether region 2 exists (B).

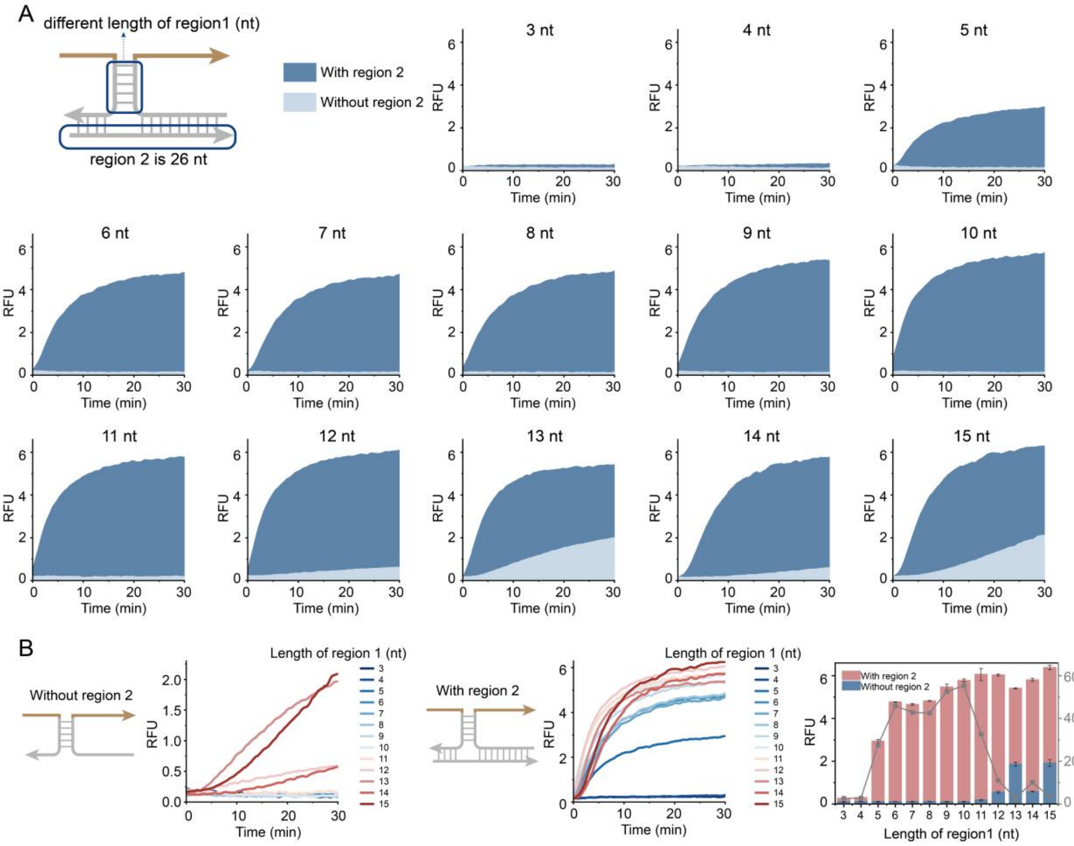

Figure S3. When region 2 is 26nt, the fluorescence curves of region 1 of different lengths (A), and the signal comparison of whether region 2 exists (B).

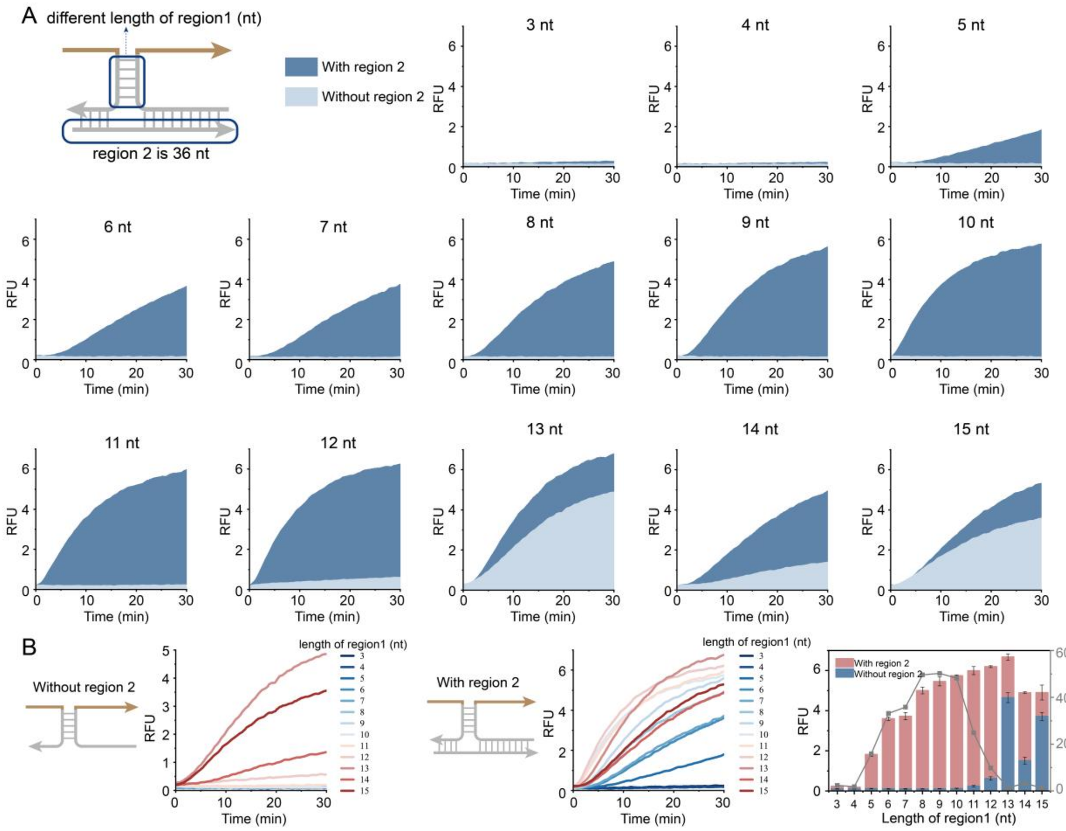

Figure S4. When region 2 is 36nt, the fluorescence curves of region 1 of different lengths (A), and the signal comparison of whether region 2 exists (B).

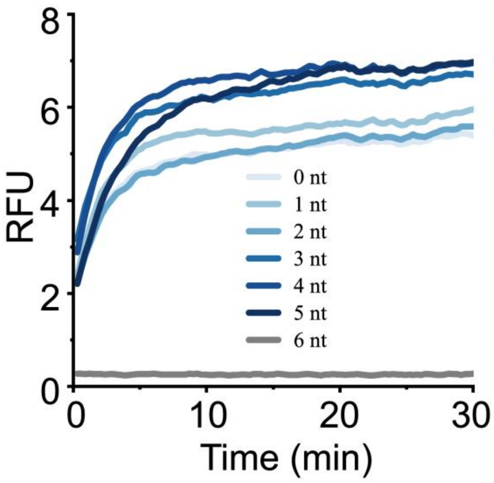

Figure S5. Fluorescence curves of different spacer lengths.

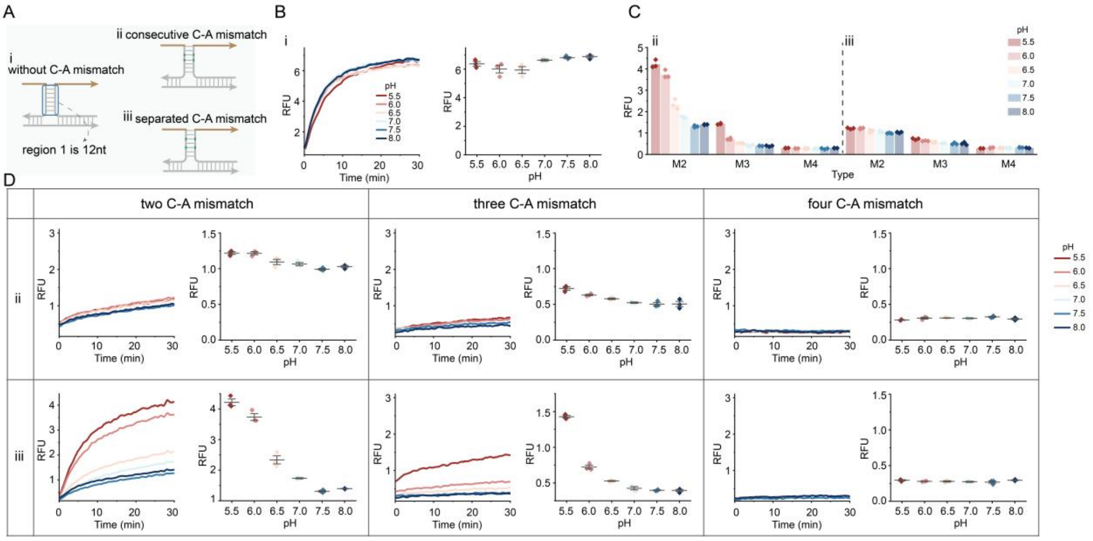

Figure S6. (A) Schematic diagram of different types of C-A mismatches with 12nt region 1. (B) Fluorescence curve of fully matched region 1 without C-A mismatches. (C) Effect of C-A mismatch arrangement and quantity on the pH-dependent signal profile. (D) Fluorescence curves of different types of C-A mismatches.

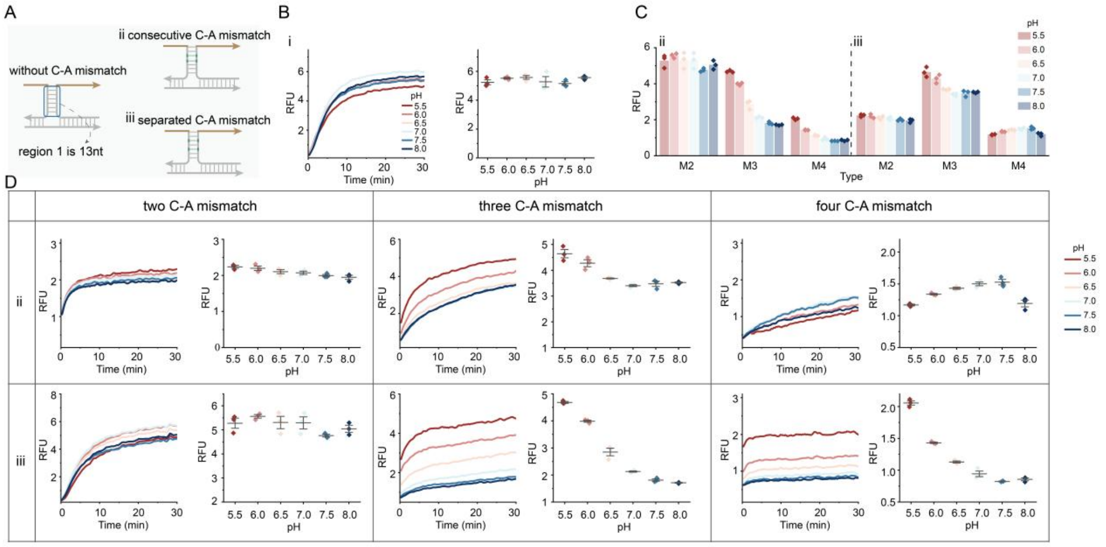

Figure S7. (A) Schematic diagram of different types of C-A mismatches with 13nt region 1. (B) Fluorescence curve of fully matched region 1 without C-A mismatches. (C) Effect of C-A mismatch arrangement and quantity on the pH-dependent signal profile. (D) Fluorescence curves of different types of C-A mismatches.

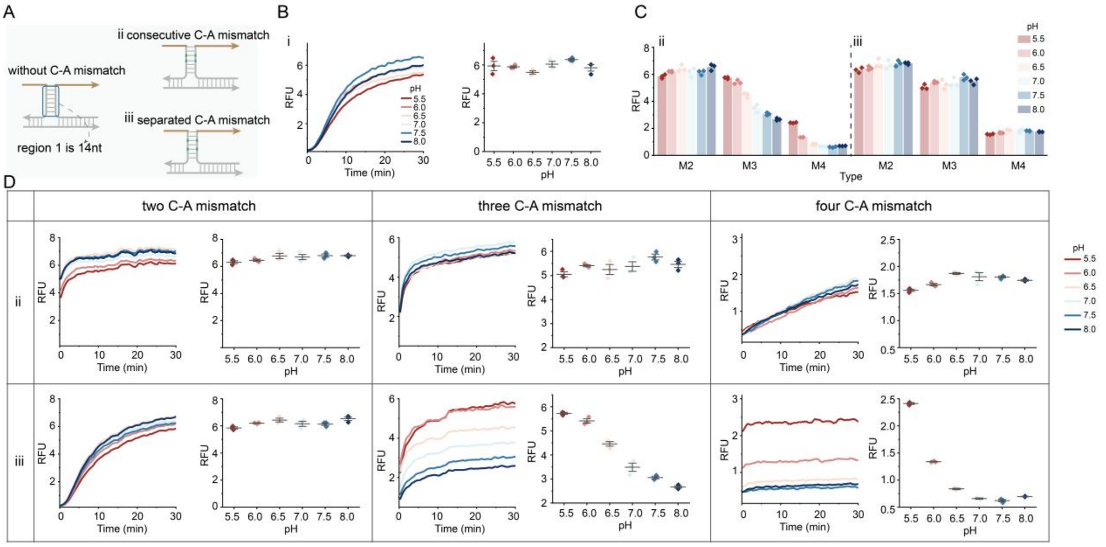

Figure S8. (A) Schematic diagram of different types of C-A mismatches with 14nt region 1. (B) Fluorescence curve of fully matched region 1 without C-A mismatches. (C) Effect of C-A mismatch arrangement and quantity on the pH-dependent signal profile. (D) Fluorescence curves of different types of C-A mismatches.

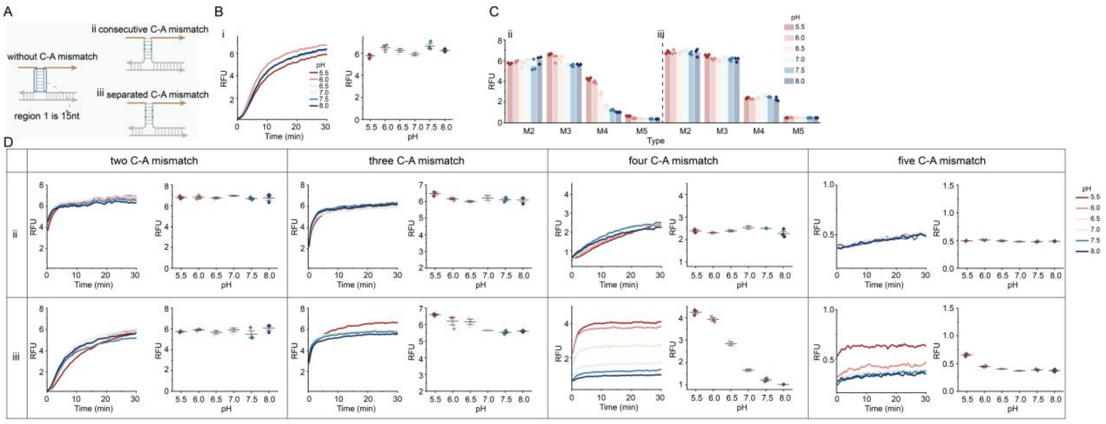

Figure S9. (A) Schematic diagram of different types of C-A mismatches with 15nt region 1. (B) Fluorescence curve of fully matched region 1 without C-A mismatches. (C) Effect of C-A mismatch arrangement and quantity on the pH-dependent signal profile. (D) Fluorescence curves of different types of C-A mismatches.

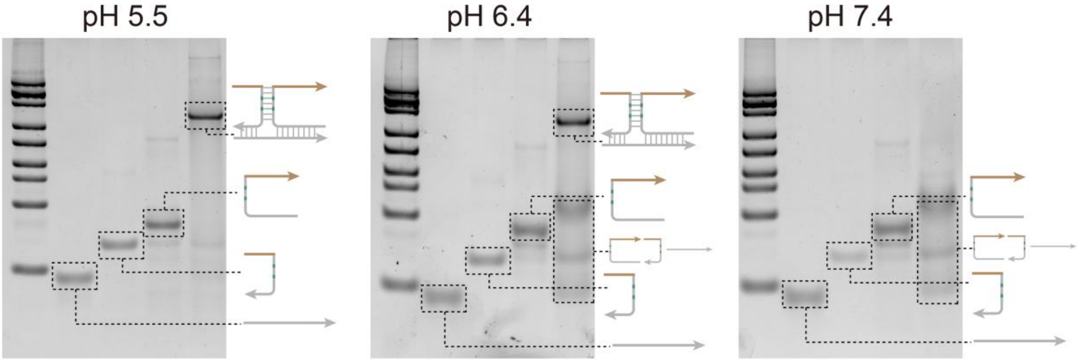

Figure S10. PAGE validation of C-A mismatch-mediated high-pH OFF module.

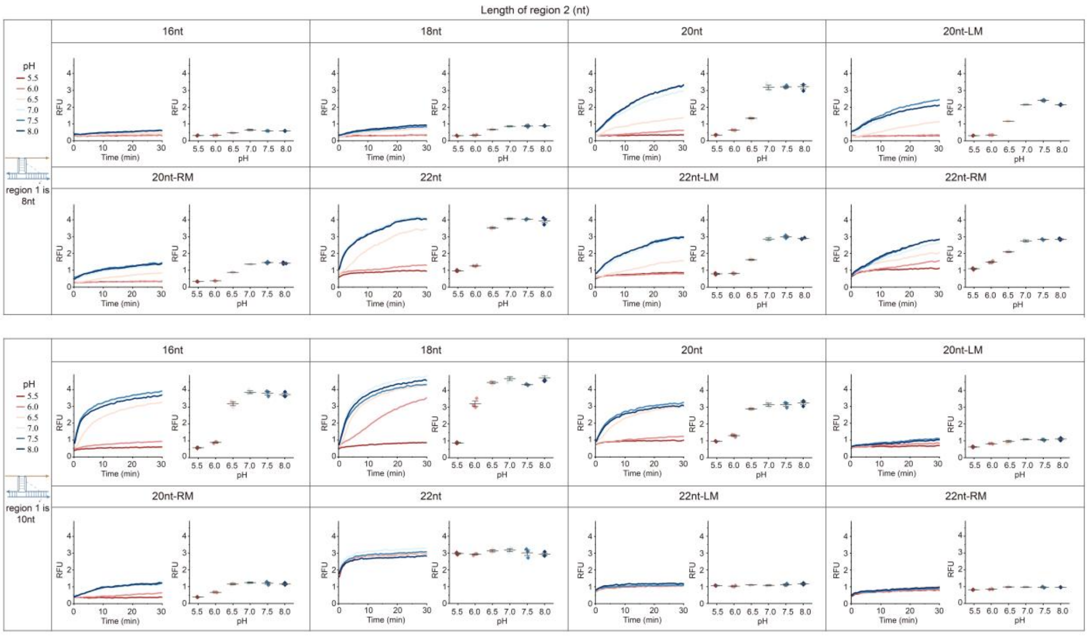

Figure S11. Fluorescence curve of C6 i-motif and framework topology (region 1: 8 vs 10 nt; region 2: 16-22 nt). LM1 refers to a mismatch on the left side of spacer in region 2, and RM1 indicates a mismatch on the right side of the spacer.

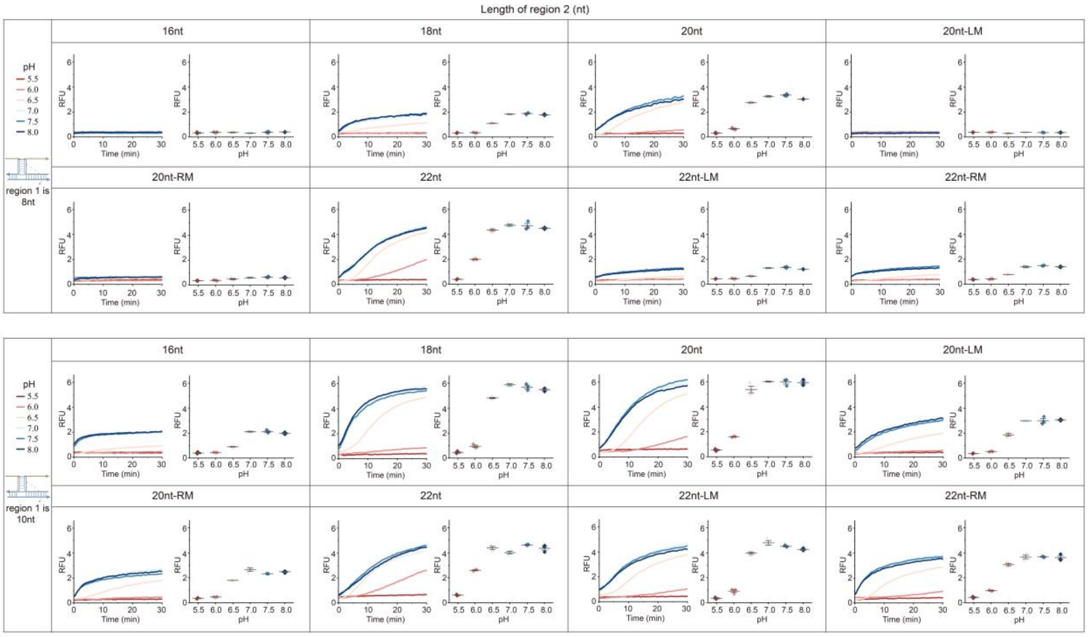

Figure S12. Fluorescence curve of C5 i-motif and framework topology (region 1: 8 vs 10 nt; region 2: 16-22 nt). LM1 refers to a mismatch on the left side of spacer in region 2, and RM1 indicates a mismatch on the right side of the spacer.

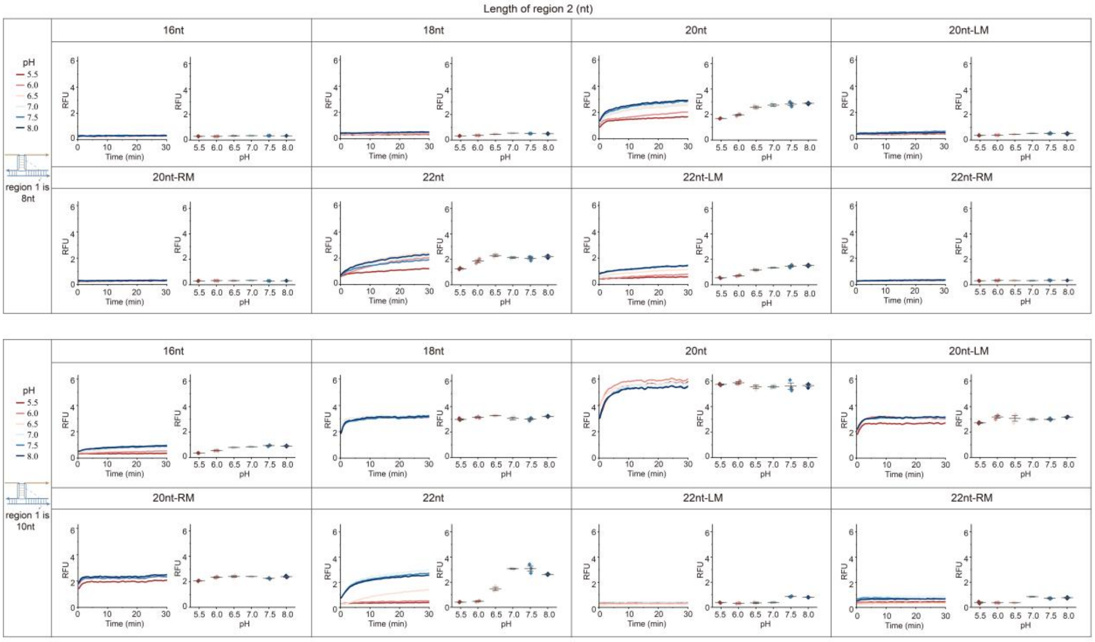

Figure S13. Fluorescence curve of C4 i-motif and framework topology (region 1: 8 vs 10 nt; region 2: 16-22 nt). LM1 refers to a mismatch on the left side of spacer in region 2, and RM1 indicates a mismatch on the right side of the spacer.

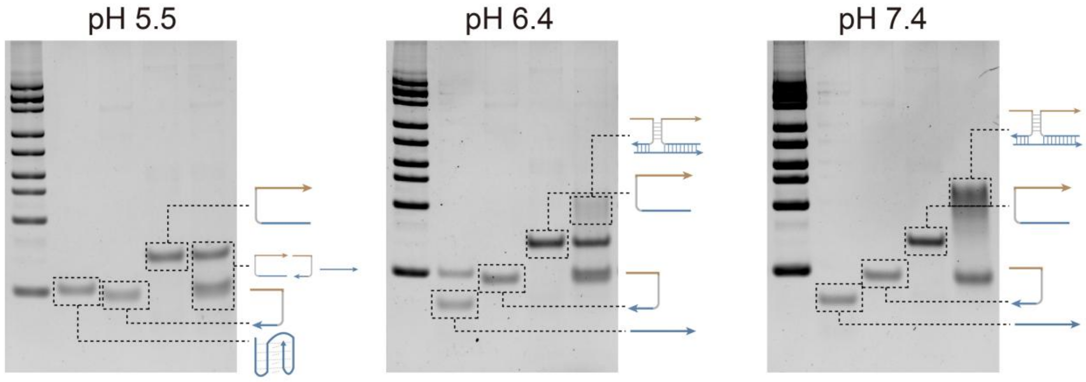

Figure S14. PAGE validation of i-motif-mediated low-pH OFF module.

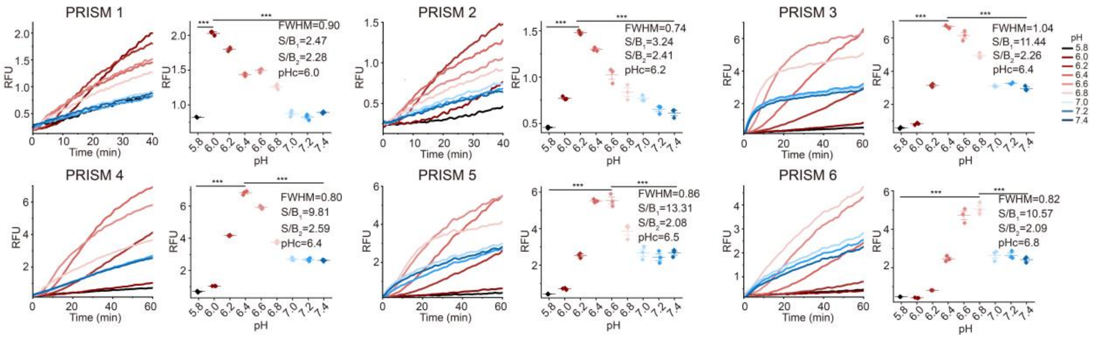

Figure S15. Different types of PRISM probes. Statistical significance between peak signal and outer boundaries (pH 5.8 and pH 7.4) was analyzed using a two-tailed Student's t-test (***denotes $p < 0.001$).

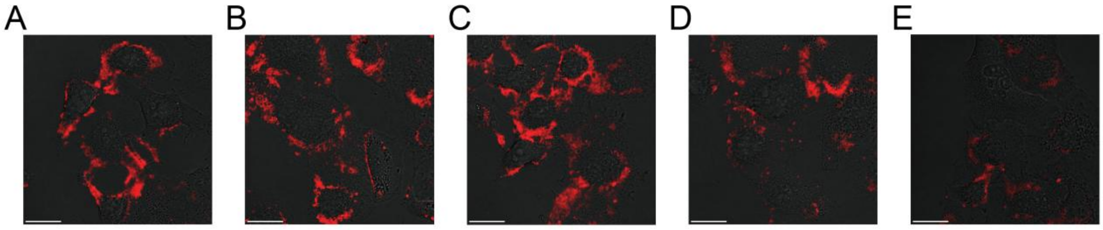

Figure S16. Confocal laser scanning microscopy (CLSM) characterization for the optimization of probe incubation time. HeLa cells were incubated with 300 nM of the c-MET-anchored PRISM probe in Phy buffer (pH 6.4) at room temperature for (A) 10 min, (B) 20 min, (C) 30 min, (D) 60 min, and (E) 120 min. Scale bars: 20 μm.

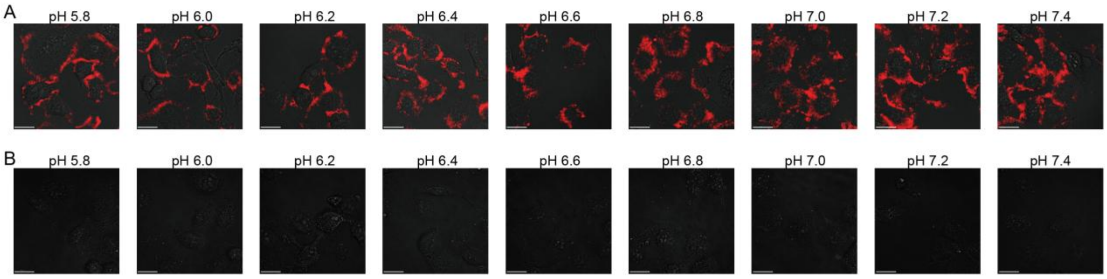

Figure S17. At different pH values, imaging results of aptamer (A) and PRISM probe with random aptamer sequences (B). Scale bars: 20 μm.

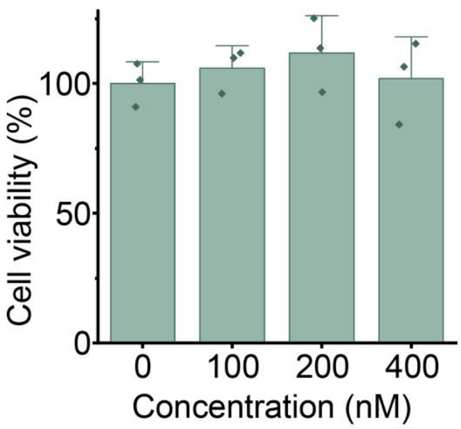

Figure S18. Cytotoxicity evaluation of the PRISM probe on HeLa cells. Cell viability was measured after 4 days (96 h) of incubation with the PRISM probe at different concentrations. The cell viability of the untreated group (0 nM) was defined as 100%.

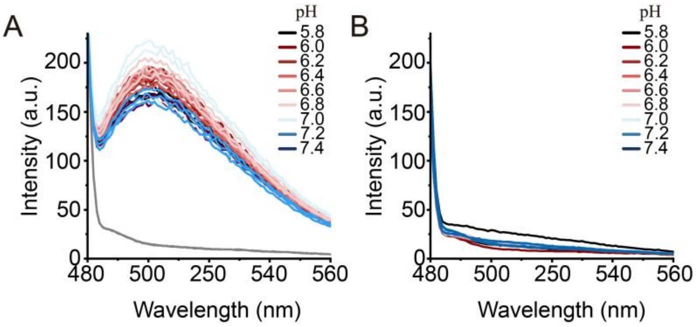

Figure S19. Fluorescence measurements of the intact DNA Lettuce (A) and the free DFHBI-1T dye (B) across a pH range of 5.5 to 8.0.

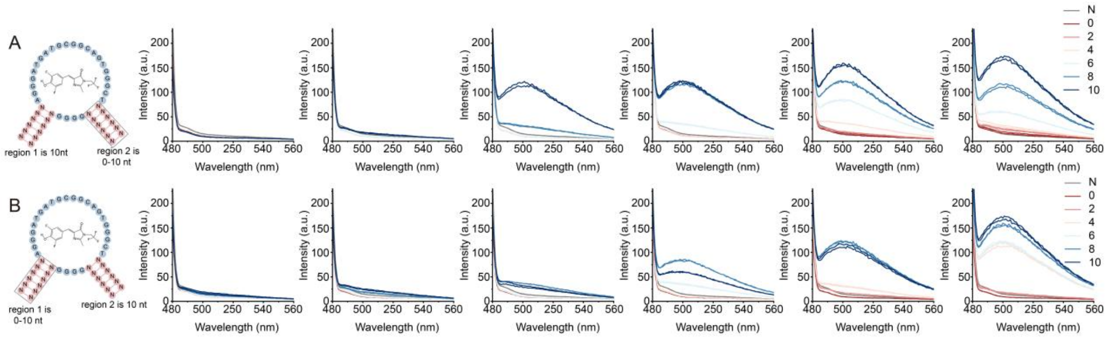

Figure S20. An orthogonal fluorescence measurements analysis of the lengths of region 2 (A) and region 1 (B).

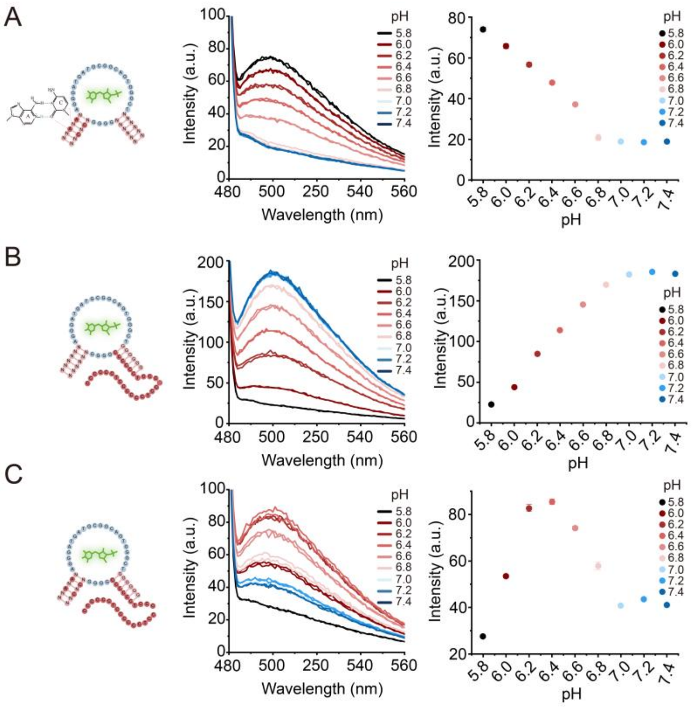

Figure S21. An fluorescence measurements of DNA Lettuce with acidic response (A), DNA Lettuce with alkaline response (B) and DNA Lettuce with narrow window pH response (C).

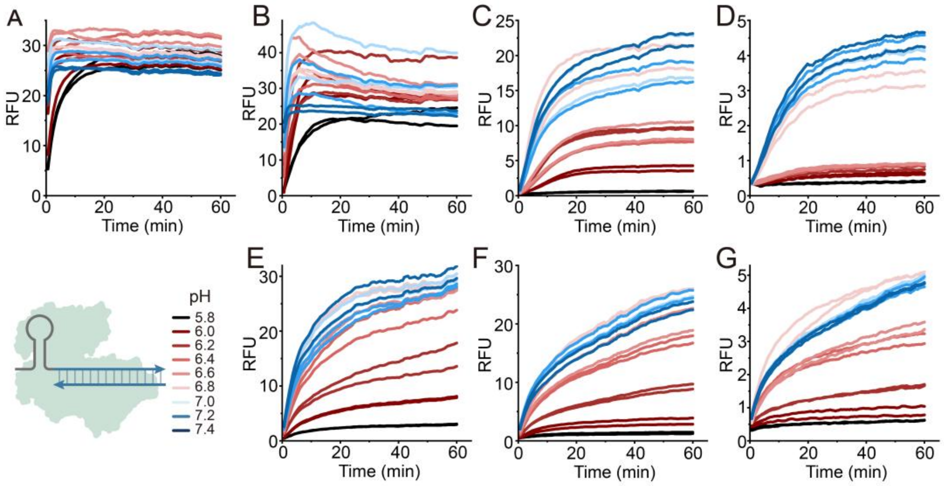

Figure S22. The curve of pH-dependent fluorescence across various concentrations of Cas enzyme and target RNA. (A) 10 nM Cas+10 nM targetRNA, (B) 2.5 nM Cas+10 nM targetRNA, (C) 1.25 nM Cas+10 nM targetRNA, (D) 0.625 nM Cas+10 nM targetRNA, (E) 10 nM Cas+2 nM targetRNA, (F) 10 nM Cas+0.4 nM targetRNA, (G) 10 nM Cas+0.08 nM targetRNA.

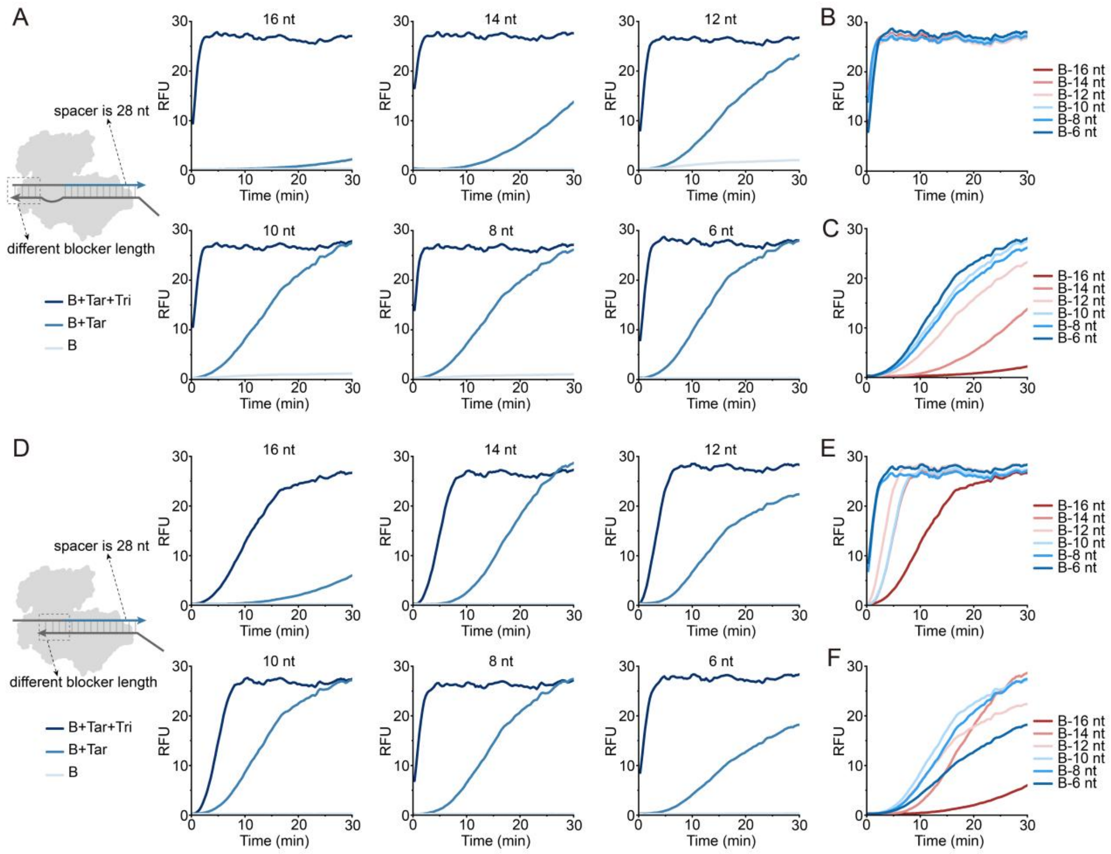

Figure S23. Fluorescence curves of blocker in the 5'-end (A-C) or 3'-end (D-F) of DR region when the spacer region of crRNA is 28nt. Comparison of trigger-induced deblocking signal (B, E, B+Tar+Tri) and the background signal (C, F, B+Tar).

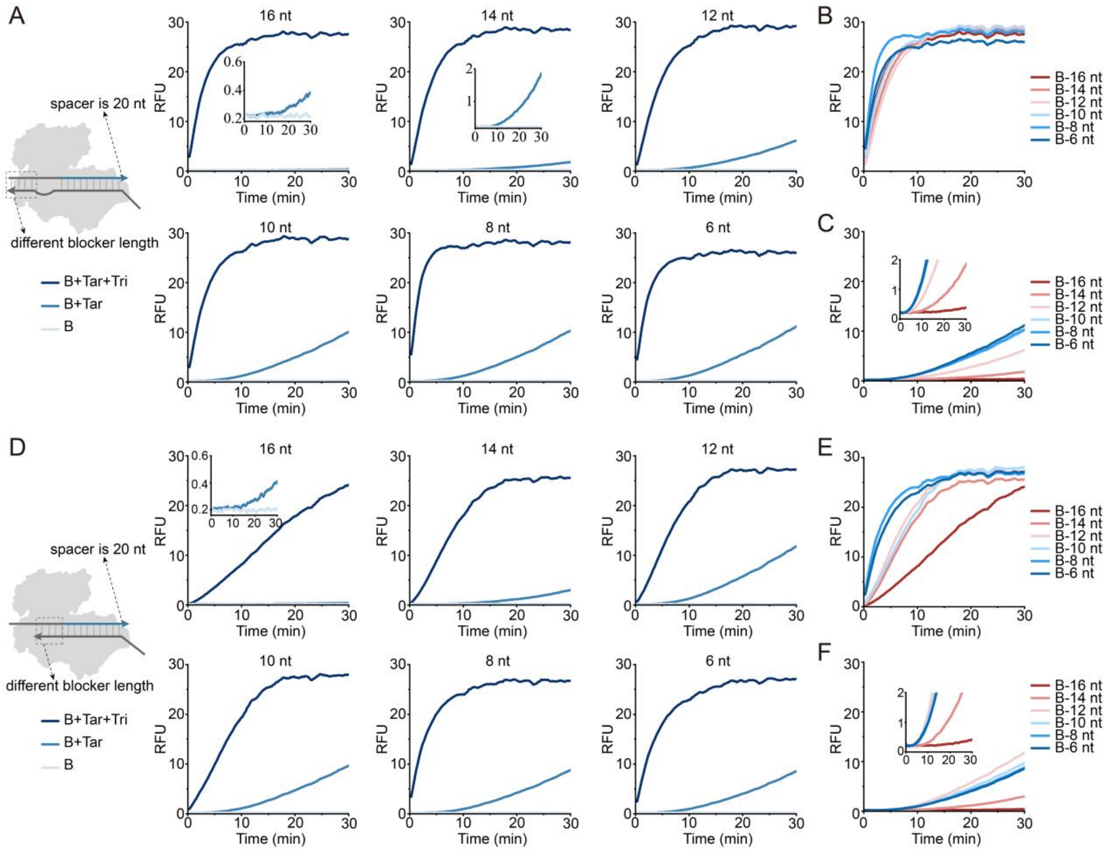

Figure S24. Fluorescence curves of blocker in the 5'-end (A-C) or 3'-end (D-F) of DR region when the spacer region of crRNA is 20nt. Comparison of trigger-induced deblocking signal (B, E, B+Tar+Tri) and the background signal (C, F, B+Tar).

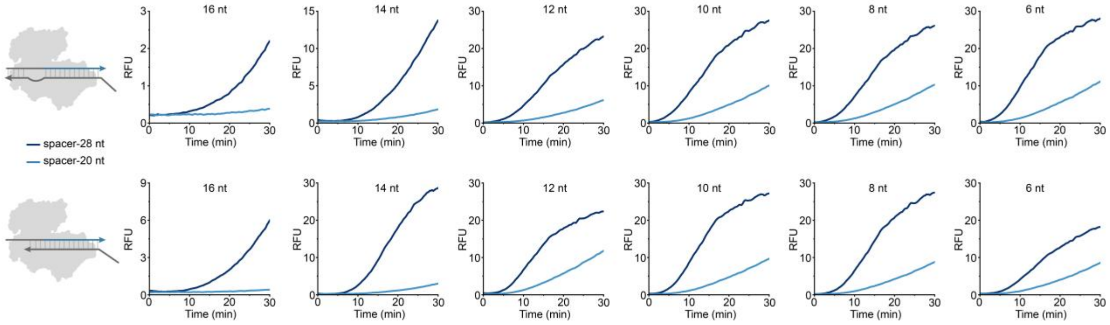

Figure S25. Comparison of spacers of crRNA with different blocker positions and lengths.

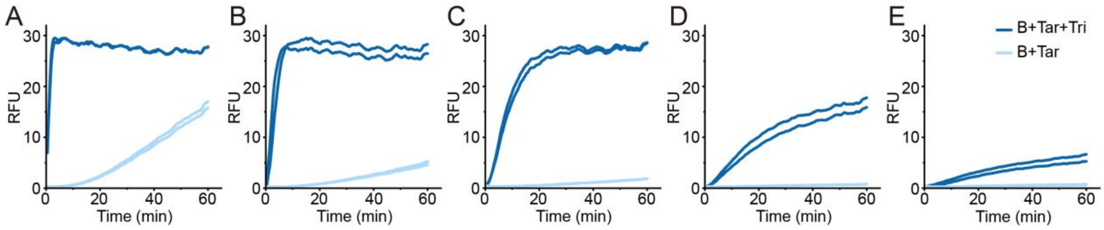

Figure S26. Fluorescence curves of target RNA concentrations at 50 nM (A), 10 nM (B), 2 nM (C), 0.4 nM (D), and 0.08 nM (E).

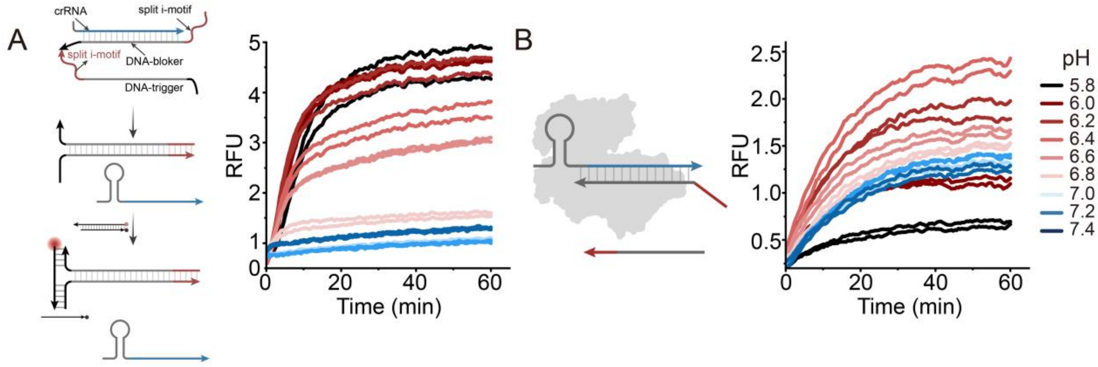

Figure S27. (A) Engineering a split i-motif into the toehold region of the TMSD reaction. (B) Synergizing the split i-motif-driven "Acidic-ON" logic with the intrinsic "Base-ON" activity of Cas enzyme.

Table S1. The sequences of DNA used in experiment. (Region 1 is bolded, and region 2 is underlined. The mismatch base is red. The C-A mismatches are green, the i-motif is blue, and the aptamer is italic.)

Name	Sequence (5'-3')
Tri-probe	TTCACATCATCGCAGGGTAGGTAGGTAGGGTCATAG
Tri-probe-1	TTCACATCAT
Tri-probe-2	CGCAGGGTAGGTAGGTAGGGTCATAG
Probe-ROX	/ROX/CTATGACCCTACCTACCTACCCTGCGATGATGTGAA
Probe-BHQ2	CGCAGGGTAGGTAGGTAGGGTCATAG/BHQ2/
$R_1$3-1	TTCACATCATTT**GTT**
$R_1$3-2	**AAC**CGCAGGGTAGGTAGGTAGGGTCATAG
$R_1$4-1	TTCACATCATTT**GGTT**
$R_1$4-2	**AACC**CGCAGGGTAGGTAGGTAGGGTCATAG
$R_1$5-1	TTCACATCATTT**AGGTT**
$R_1$5-2	**AACCT**CGCAGGGTAGGTAGGTAGGGTCATAG
$R_1$6-1	TTCACATCATTT**AGGTTC**
$R_1$6-2	**GAACCT**CGCAGGGTAGGTAGGTAGGGTCATAG
$R_1$7-1	TTCACATCATTT**AAGGTTC**
$R_1$7-2	**GAACCTT**CGCAGGGTAGGTAGGTAGGGTCATAG
$R_1$8-1	TTCACATCATTT**CTAAGGTT**
$R_1$8-2	**AACCTTAG**CGCAGGGTAGGTAGGTAGGGTCATAG
$R_1$9-1	TTCACATCATTT**CTAAGGTTC**
$R_1$9-2	**GAACCTTAG**CGCAGGGTAGGTAGGTAGGGTCATAG
$R_1$10-1	TTCACATCATTT**CTAAGGTTCA**
$R_1$10-2	**TGAACCTTAG**CGCAGGGTAGGTAGGTAGGGTCATAG
$R_1$11-1	TTCACATCATTT**CTAAGGTTCAC**
$R_1$11-2	**GTGAACCTTAG**CGCAGGGTAGGTAGGTAGGGTCATAG
$R_1$12-1	TTCACATCATTT**CTAAGGTTCACA**
$R_1$12-2	**TGTGAACCTTAG**CGCAGGGTAGGTAGGTAGGGTCATAG
$R_1$13-1	TTCACATCATTT**CCTAAGGTTCACA**
$R_1$13-2	**TGTGAACCTTAGG**CGCAGGGTAGGTAGGTAGGGTCATA G
$R_1$14-1	TTCACATCATTT**CACTAAGGTTCACA**
$R_1$14-2	**TGTGAACCTTAGTG**CGCAGGGTAGGTAGGTAGGGTCAT AG
$R_1$15-1	TTCACATCATTT**CAACTAAGGTTCACA**

$R_1$15-2	**TGTGAACCTTAGTTG**CGCAGGGTAGGTAGGTAGGGTCATAG
$R_2$18	AGCGGTTGATTTTTGTTGGGC
$R_1$11-1-mismatch1	TTCACATCATTT**CCTAAGCTTCACA**
$R_1$11-1-mismatch2	TTCACATCATTT**CCTCAGCTTCACA**
$R_1$11-1-mismatch3	TTCACATCATTT**CCTCAGCTACACA**
$R_1$3$R_2$18-1	TTCACATCATTT**GTT**TCAACCGCT
$R_1$3$R_2$18-2	GCCCAACAAAACCGCAGGGTAGGTAGGTAGGGTCATAG
$R_1$4$R_2$18-1	TTCACATCATTT**GGTT**TCAACCGCT
$R_1$4$R_2$18-2	GCCCAACAA**AACC**CGCAGGGTAGGTAGGTAGGGTCATAG
$R_1$5$R_2$18-1	TTCACATCATTT**AGGTT**TCAACCGCT
$R_1$5$R_2$18-2	GCCCAACAA**AACCT**CGCAGGGTAGGTAGGTAGGGTCATAG
$R_1$6$R_2$18-1	TTCACATCATTT**AGGTTC**TCAACCGCT
$R_1$6$R_2$18-2	GCCCAACAA**GAACCT**CGCAGGGTAGGTAGGTAGGGTCATAG
$R_1$7$R_2$18-1	TTCACATCATTT**AAGGTTC**TCAACCGCT
$R_1$7$R_2$18-2	GCCCAACAA**GAACCTT**CGCAGGGTAGGTAGGTAGGGTCATAG
$R_1$8$R_2$18-1	TTCACATCATTT**CTAAGGTT**TCAACCGCT
$R_1$8$R_2$18-2	GCCCAACAA**AACCTTAG**CGCAGGGTAGGTAGGTAGGGTCATAG
$R_1$9$R_2$18-1	TTCACATCATTT**CTAAGGTTC**TCAACCGCT
$R_1$9$R_2$18-2	GCCCAACAA**GAACCTTAG**CGCAGGGTAGGTAGGTAGGGTCATAG
$R_1$10$R_2$18-1	TTCACATCATTT**CTAAGGTTCA**TCAACCGCT
$R_1$10$R_2$18-2	GCCCAACAA**TGAACCTTAG**CGCAGGGTAGGTAGGTAGGGTCATAG
$R_1$11$R_2$18-1	TTCACATCATTT**CTAAGGTTCAC**TCAACCGCT
$R_1$11$R_2$18-2	GCCCAACAA**GTGAACCTTAG**CGCAGGGTAGGTAGGTAGGGTCATAG
$R_1$12$R_2$18-1	TTCACATCATTT**CTAAGGTTCACA**TCAACCGCT
$R_1$12$R_2$18-2	GCCCAACAA**TGTGAACCTTAG**CGCAGGGTAGGTAGGTAGGGTCATAG
$R_1$13$R_2$18-1	TTCACATCATTT**CCTAAGGTTCACA**TCAACCGCT
$R_1$13$R_2$18-2	GCCCAACAA**TGTGAACCTTAGG**CGCAGGGTAGGTAGGTAGGGTCATAG
$R_1$14$R_2$18-1	TTCACATCATTT**CACTAAGGTTCACA**TCAACCGCT

$R_1$14$R_2$18-2	GCCCAACAA**TGTGAACCTTAGTG**CGCAGGGTAGGTAG GTAGGGTCATAG
$R_1$15$R_2$18-1	TTCACATCATTT**CAACTAAGGTTCACA**TCAACCGCT
$R_1$15$R_2$18-2	GCCCAACAA**TGTGAACCTTAGTTG**CGCAGGGTAGGTAG GTAGGGTCATAG
$R_2$26	AAAGAGCGGTTGATATTTGTTGGGCGGCT
$R_1$3$R_2$26-1	TTCACATCATTT**GTT**TCAACCGCTCTTT
$R_1$3$R_2$26-2	AGCCGCCCAACAA**AAC**CGCAGGGTAGGTAGGTAGGGTC ATAG
$R_1$4$R_2$26-1	TTCACATCATTT**GGTT**TCAACCGCTCTTT
$R_1$4$R_2$26-2	AGCCGCCCAACAA**AACC**CGCAGGGTAGGTAGGTAGGGT CATAG
$R_1$5$R_2$26-1	TTCACATCATTTA**GGTT**TCAACCGCTCTTT
$R_1$5$R_2$26-2	AGCCGCCCAACAA**AACCT**CGCAGGGTAGGTAGGTAGGG TCATAG
$R_1$6$R_2$26-1	TTCACATCATTT**AGGTTC**TCAACCGCTCTTT
$R_1$6$R_2$26-2	AGCCGCCCAACAA**GAACCT**CGCAGGGTAGGTAGGTAGG GTCATAG
$R_1$7$R_2$26-1	TTCACATCATTT**AAGGTTC**TCAACCGCTCTTT
$R_1$7$R_2$26-2	AGCCGCCCAACAA**GAACCTT**CGCAGGGTAGGTAGGTAG GGTCATAG
$R_{26}R_2$26-1	TTCACATCATTT**CTAAGGTT**TCAACCGCTCTTT
$R_{26}R_2$26-2	AGCCGCCCAACAA**AACCTTAG**CGCAGGGTAGGTAGGTA GGGTCATAG
$R_1$9$R_2$26-1	TTCACATCATTT**CTAAGGTTC**TCAACCGCTCTTT
$R_1$9$R_2$26-2	AGCCGCCCAACAA**GAACCTTAG**CGCAGGGTAGGTAGGT AGGGTCATAG
$R_1$10$R_2$26-1	TTCACATCATTT**CTAAGGTTCA**TCAACCGCTCTTT
$R_1$10$R_2$26-2	AGCCGCCCAACAA**TGAACCTTAG**CGCAGGGTAGGTAGG TAGGGTCATAG
$R_1$11$R_2$26-1	TTCACATCATTT**CTAAGGTTCAC**TCAACCGCTCTTT
$R_1$11$R_2$26-2	AGCCGCCCAACAA**GTGAACCTTAG**CGCAGGGTAGGTAG GTAGGGTCATAG
$R_1$12$R_2$26-1	TTCACATCATTT**CTAAGGTTCACA**TCAACCGCTCTTT
$R_1$12$R_2$26-2	AGCCGCCCAACAA**TGTGAACCTTAG**CGCAGGGTAGGTA GGTAGGGTCATAG
$R_1$13$R_2$26-1	TTCACATCATTT**CCTAAGGTTCACA**TCAACCGCTCTTT
$R_1$13$R_2$26-2	AGCCGCCCAACAA**TGTGAACCTTAGG**CGCAGGGTAGGT

	AGGTAGGGTCATAG
$R_{1}14R_{2}26$-1	TTCACATCATTT**CACTAAGGTTCACA**TCAACCGCTCTTT
$R_{1}14R_{2}26$-2	AGCCGCCCAACAA**TGTGAACCTTAGTG**CGCAGGGTAGGTAGGTAGGGTCATAG
$R_{1}15R_{2}26$-1	TTCACATCATTT**CAACTAAGGTTCACA**TCAACCGCTCTTT
$R_{1}15R_{2}26$-2	AGCCGCCCAACAA**TGTGAACCTTAGTTG**CGCAGGGTAGGTAGGTAGGGTCATAG
$R_{2}36$	GCTCCAAAGAGCGGTTGATATTTGTTGGGCGGCTCAGCT
$R_{1}3R_{2}36$-1	TTCACATCATTT**GTT**TCAACCGCTCTTTGGAGC
$R_{1}3R_{2}36$-2	AGCTGAGCCGCCCAACAA**AAC**CGCAGGGTAGGTAGGTAGGGTCATAG
$R_{1}4R_{2}36$-1	TTCACATCATTT**GGTT**TCAACCGCTCTTTGGAGC
$R_{1}4R_{2}36$-2	AGCTGAGCCGCCCAACAA**AACC**CGCAGGGTAGGTAGGTAGGGTCATAG
$R_{1}5R_{2}36$-1	TTCACATCATTT**AGGTT**TCAACCGCTCTTTGGAGC
$R_{1}5R_{2}36$-2	AGCTGAGCCGCCCAACAA**AACCT**CGCAGGGTAGGTAGGTAGGGTCATAG
$R_{1}6R_{2}36$-1	TTCACATCATTT**AGGTTC**TCAACCGCTCTTTGGAGC
$R_{1}6R_{2}36$-2	AGCTGAGCCGCCCAACAA**GAACCT**CGCAGGGTAGGTAGGTAGGGTCATAG
$R_{1}7R_{2}36$-1	TTCACATCATTT**AAGGTTC**TCAACCGCTCTTTGGAGC
$R_{1}7R_{2}36$-2	AGCTGAGCCGCCCAACAA**GAACCTT**CGCAGGGTAGGTAGGTAGGGTCATAG
$R_{1}8R_{2}36$-1	TTCACATCATTT**CTAAGGTT**TCAACCGCTCTTTGGAGC
$R_{1}8R_{2}36$-2	AGCTGAGCCGCCCAACAA**AACCTTAG**CGCAGGGTAGGTAGGTAGGGTCATAG
$R_{1}9R_{2}36$-1	TTCACATCATTT**CTAAGGTTC**TCAACCGCTCTTTGGAGC
$R_{1}9R_{2}36$-2	AGCTGAGCCGCCCAACAA**GAACCTTAG**CGCAGGGTAGGTAGGTAGGGTCATAG
$R_{1}10R_{2}36$-1	TTCACATCATTT**CTAAGGTTCA**TCAACCGCTCTTTGGAGC
$R_{1}10R_{2}36$-2	AGCTGAGCCGCCCAACAA**TGAACCTTAG**CGCAGGGTAGGTAGGTAGGGTCATAG
$R_{1}11R_{2}36$-1	TTCACATCATTT**CTAAGGTTCAC**TCAACCGCTCTTTGGAGC
$R_{1}11R_{2}36$-2	AGCTGAGCCGCCCAACAA**GTGAACCTTAG**CGCAGGGTAGGTAGGTAGGGTCATAG
$R_{1}12R_{2}36$-1	TTCACATCATTT**CTAAGGTTCACA**TCAACCGCTCTTTGGAGC
$R_{1}12R_{2}36$-2	AGCTGAGCCGCCCAACAA**TGTGAACCTTAG**CGCAGGGT

	AGGTAGGTAGGGTCATAG
R$_1$13R$_2$36-1	TTCACATCATTT**CCTAAGGTTCACA**TCAACCGCTCTTTGGAGC
R$_1$13R$_2$36-2	AGCTGAGCCGCCCAACAA**TGTGAACCTTAGG**CGCAGGGTAGGTAGGTAGGGTCATAG
R$_1$14R$_2$36-1	TTCACATCATTT**CACTAAGGTTCACA**TCAACCGCTCTTTGGAGC
R$_1$14R$_2$36-2	AGCTGAGCCGCCCAACAA**TGTGAACCTTAGTG**CGCAGGGTAGGTAGGTAGGGTCATAG
R$_1$15R$_2$36-1	TTCACATCATTT**CAACTAAGGTTCACA**TCAACCGCTCTTTGGAGC
R$_1$15R$_2$36-2	AGCTGAGCCGCCCAACAA**TGTGAACCTTAGTTG**CGCAGGGTAGGTAGGTAGGGTCATAG
R$_2$26-spacer0	AAAGAGCGGTTGATTGTTGGGCGGCT
R$_2$26-spacer1	AAAGAGCGGTTGATTTGTTGGGCGGCT
R$_2$26-spacer2	AAAGAGCGGTTGATTTTGTTGGGCGGCT
R$_2$26-spacer4	AAAGAGCGGTTGATATATTGTTGGGCGGCT
R$_2$26-spacer5	AAAGAGCGGTTGATATATTTGTTGGGCGGCT
R$_1$13R$_2$26-1-mis2	TTCACATCATTT**CCTACGGTACACA**TCAACCGCTCTTT
R$_1$13R$_2$26-1-mis3	TTCACATCATTT**CCAAACGTACACA**TCAACCGCTCTTT
R$_1$13R$_2$26-1-mis4	TTCACATCATTT**CCAAACGTACCCA**TCAACCGCTCTTT
R$_2$26-mis1	AAAGAGCCGTTGATATTTGTTGGGCGGCT
R$_2$26-mis2	AAAGAGCCGTTGATATTTGTTGCGCGGCT
R$_2$26-mis3	AAAGAGCGGTTGATATTTGTTGCGCGGCT
R$_2$26-mis4	AAACAGCGCTTGATATTTGTTGCGCGGCT
R$_2$26-mis5	AAAGAGCCGTTGATATTTGTTCGGCCGCT
R$_1$12R$_2$26-2-2CA$_{sp}$	AGCCGCCCAACAA**TGCGAACCCTAG**CGCAGGGTAGGTAGGTAGGGTCATAG
R$_1$13R$_2$26-2-2CA$_{sp}$	AGCCGCCCAACAA**TGTAAACCTCAGG**CGCAGGGTAGGTAGGTAGGGTCATAG
R$_1$14R$_2$26-2-2CA$_{sp}$	AGCCGCCCAACAA**TGTAAACCTCAGTG**CGCAGGGTAGGTAGGTAGGGTCATAG
R$_1$15R$_2$26-2-2CA$_{sp}$	AGCCGCCCAACAA**TGTAAACCTTAATTG**CGCAGGGTAGGTAGGTAGGGTCATAG
R$_1$12R$_2$26-1-2CA$_{con}$	TTCACATCATTT**CTAAAATTCACA**TCAACCGCTCTTT
R$_1$13R$_2$26-1-2CA$_{con}$	TTCACATCATTT**CCTAAAATTCACA**TCAACCGCTCTTT
R$_1$14R$_2$26-1-2CA$_{con}$	TTCACATCATTT**CACTAAAATTCACA**TCAACCGCTCTTT
R$_1$15R$_2$26-1-2CA$_{con}$	TTCACATCATTT**CAACTAAAATTCACA**TCAACCGCTCTTT

$R_1$12$R_2$26-1-3CA$_{sp}$	TTCACATCATTT**CCAAAGTCCACA**TCAACCGCTCTTT
$R_1$13$R_2$26-1-3CA$_{sp}$	TTCACATCATTT**CCCAAAGTCCACA**TCAACCGCTCTTT
$R_1$14$R_2$26-1-3CA$_{sp}$	TTCACATCATTT**CACCAAAGTCCACA**TCAACCGCTCTTT
$R_1$15$R_2$26-1-3CA$_{sp}$	TTCACATCATTT**CAACCAAAGTCCACA**TCAACCGCTCTTT
$R_1$12$R_2$26-1-3CA$_{con}$	TTCACATCATTT**CTAAGACCCACA**TCAACCGCTCTTT
$R_1$13$R_2$26-1-3CA$_{con}$	TTCACATCATTT**CCTAAGACCCACA**TCAACCGCTCTTT
$R_1$14$R_2$26-1-3CA$_{con}$	TTCACATCATTT**CACTAAAACTCACA**TCAACCGCTCTTT
$R_1$15$R_2$26-1-3CA$_{con}$	TTCACATCATTT**CAACTAAAACTCACA**TCAACCGCTCTTT
$R_1$12$R_2$26-2-4CA$_{sp}$	AGCCGCCCAACAA**TATAAACCCTAA**CGCAGGGTAGGTAGGTAGGGTCATAG
$R_1$13$R_2$26-2-4CA$_{sp}$	AGCCGCCCAACAA**TATAAACCTCAAG**CGCAGGGTAGGTAGGTAGGGTCATAG
$R_1$14$R_2$26-2-4CA$_{sp}$	AGCCGCCCAACAA**TATAAACCCTAATG**CGCAGGGTAGGTAGGTAGGGTCATAG
$R_1$15$R_2$26-2-4CA$_{sp}$	AGCCGCCCAACAA**TATAAACCCTAGTCG**CGCAGGGTAGGTAGGTAGGGTCATAG
$R_1$12$R_2$26-1-4CA$_{con}$	TTCACATCATTT**CTAAAACCCACA**TCAACCGCTCTTT
$R_1$13$R_2$26-1-4CA$_{con}$	TTCACATCATTT**CCTAAAACCCACA**TCAACCGCTCTTT
$R_1$14$R_2$26-1-4CA$_{con}$	TTCACATCATTT**CACTAAAACCCACA**TCAACCGCTCTTT
$R_1$15$R_2$26-1-4CA$_{con}$	TTCACATCATTT**CAACTAAAACCCACA**TCAACCGCTCTTT
$R_1$15$R_2$26-2-5CA$_{sp}$	AGCCGCCCAACAA**TATAAACCCTAATCG**CGCAGGGTAGGTAGGTAGGGTCATAG
$R_1$15$R_2$26-1-5CA$_{con}$	TTCACATCATTT**CAACTAAAACCCACA**TCAACCGCTCTTT
$R_1$15$R_2$26-2-5CA$_{con}$	AGCCGCCCAACAA**TGTGAACCCTAGTTG**CGCAGGGTAGGTAGGTAGGGTCATAG
$R_1$12$R_2$26-1-reset	/ROX/**CTAAGGTTCACA**TCAACCGCTCTTT
$R_1$12$R_2$26-2-reset	AGCCGCCCAACAA**TGCGAACCCTAG**/BHQ2/
$R_1$14$R_2$26-1-G	TTCACATCATTT**CACGAAGGTGCACA**TCAACCGCTCTTT
$R_1$14$R_2$26-1-T	TTCACATCATTT**CACTAATGTTCACA**TCAACCGCTCTTT
$R_1$14$R_2$26-1-A	TTCACATCATTT**CACAAAAGTACACA**TCAACCGCTCTTT
$R_1$14$R_2$26-1-C	TTCACATCATTT**CACCAACGTCCACA**TCAACCGCTCTTT
$R_1$14$R_2$26-2-G	AGCCGCCCAACAA**TGTGGACGTTGGTG**CGCAGGGTAGGTAGGTAGGGTCATAG
$R_1$14$R_2$26-2-T	AGCCGCCCAACAA**TGTGTACTTTTGTG**CGCAGGGTAGGTAGGTAGGGTCATAG
$R_1$14$R_2$26-2-A	AGCCGCCCAACAA**TGTGAACATTAGTG**CGCAGGGTAG

	GTAGGTAGGGTCATAG
$R_1$14$R_2$26-2-C	AGCCGCCCAACAA**TGTGCACCTTCGTG**CGCAGGGTAG GTAGGTAGGGTCATAG
$R_{2\text{-imotif-C6}}$	CCCCCCTCCCCCCTCCCCCCTCCCCCC
$R_1$8$R_2$16-1-C6	TTCACATCATTT**CTAAGGTT**GGGGGGAG
$R_1$8$R_2$16-2-C6	GAGGGGGG**AACCTTAG**CGCAGGGTAGGTAGGTAGGGTC ATAG
$R_1$8$R_2$18-1-C6	TTCACATCATTT**CTAAGGTT**GGGGGGAGG
$R_1$8$R_2$18-2-C6	GGAGGGGGG**AACCTTAG**CGCAGGGTAGGTAGGTAGGGT CATAG
$R_1$8$R_2$20-1-C6	TTCACATCATTT**CTAAGGTT**GGGGGGAGGG
$R_1$8$R_2$20-2-C6	GGGAGGGGGG**AACCTTAG**CGCAGGGTAGGTAGGTAGG GTCATAG
$R_1$8$R_2$20-1-C6-mis	TTCACATCATTT**CTAAGGTT**GGGTGGAGGG
$R_1$8$R_2$20-2-C6-mis	GGGAGGTGGG**AACCTTAG**CGCAGGGTAGGTAGGTAGGG TCATAG
$R_1$8$R_2$22-1-C6	TTCACATCATTT**CTAAGGTT**GGGGGGAGGGG
$R_1$8$R_2$22-2-C6	GGGGAGGGGGG**AACCTTAG**CGCAGGGTAGGTAGGTAG GGTCATAG
$R_1$8$R_2$22-1-C6-mis	TTCACATCATTT**CTAAGGTT**GGGGTGAGGGG
$R_1$8$R_2$22-2-C6-mis	GGGGAGTGGGG**AACCTTAG**CGCAGGGTAGGTAGGTAGG GTCATAG
$R_1$10$R_2$16-1-C6	TTCACATCATTT**CTAAGGTTCA**GGGGGGAG
$R_1$10$R_2$16-2-C6	GAGGGGGG**TGAACCTTAG**CGCAGGGTAGGTAGGTAGG GTCATAG
$R_1$10$R_2$18-1-C6	TTCACATCATTT**CTAAGGTTCA**GGGGGGAGG
$R_1$10$R_2$18-2-C6	GGAGGGGGG**TGAACCTTAG**CGCAGGGTAGGTAGGTAG GGTCATAG
$R_1$10$R_2$20-1-C6	TTCACATCATTT**CTAAGGTTCA**GGGGGGAGGG
$R_1$10$R_2$20-2-C6	GGGAGGGGGG**TGAACCTTAG**CGCAGGGTAGGTAGGTA GGGTCATAG
$R_1$10$R_2$20-1-C6-mis	TTCACATCATTT**CTAAGGTTCA**GGGTGGAGGG
$R_1$10$R_2$20-2-C6-mis	GGGAGGTGGG**TGAACCTTAG**CGCAGGGTAGGTAGGTAG GGTCATAG
$R_1$10$R_2$22-1-C6	TTCACATCATTT**CTAAGGTTCA**GGGGGGAGGGG
$R_1$10$R_2$22-2-C6	GGGGAGGGGGG**TGAACCTTAG**CGCAGGGTAGGTAGGT AGGGTCATAG
$R_1$10$R_2$22-1-C6-mis	TTCACATCATTT**CTAAGGTTCA**GGGGTGAGGGG
$R_1$10$R_2$22-2-C6-mis	GGGGAGTGGGG**TGAACCTTAG**CGCAGGGTAGGTAGGTA GGGTCATAG

$R_{2\text{-imotif-C5}}$	CCCCCTTTCCCCCTTTCCCCCTTTCCCCC
$R_1$8$R_2$16-1-C5	TTCACATCATTT**CTAAGGTT**GGGGGAAA
$R_1$8$R_2$16-2-C5	AAAGGGGG**AACCTTAG**CGCAGGGTAGGTAGGTAGGGTCATAG
$R_1$8$R_2$18-1-C5	TTCACATCATTT**CTAAGGTT**GGGGGAAAG
$R_1$8$R_2$18-2-C5	GAAAGGGGG**AACCTTAG**CGCAGGGTAGGTAGGTAGGGTCATAG
$R_1$8$R_2$20-1-C5	TTCACATCATTT**CTAAGGTT**GGGGGAAAGG
$R_1$8$R_2$20-2-C5	GGAAAGGGGG**AACCTTAG**CGCAGGGTAGGTAGGTAGGGTCATAG
$R_1$8$R_2$20-1-C5-mis	TTCACATCATTT**CTAAGGTT**GGGTGAAAGG
$R_1$8$R_2$20-2-C5-mis	GGAAAGTGGG**AACCTTAG**CGCAGGGTAGGTAGGTAGGGTCATAG
$R_1$8$R_2$22-1-C5	TTCACATCATTT**CTAAGGTT**GGGGGAAAGGG
$R_1$8$R_2$22-2-C5	GGGAAAGGGGG**AACCTTAG**CGCAGGGTAGGTAGGTAGGGTCATAG
$R_1$8$R_2$22-1-C5-mis	TTCACATCATTT**CTAAGGTT**GGGTGAAAGGG
$R_1$8$R_2$22-2-C5-mis	GGGAAAGTGGG**AACCTTAG**CGCAGGGTAGGTAGGTAGGGTCATAG
$R_1$10$R_2$16-1-C5	TTCACATCATTT**CTAAGGTTCA**GGGGGAAA
$R_1$10$R_2$16-2-C5	AAAGGGGG**TGAACCTTAG**CGCAGGGTAGGTAGGTAGGGTCATAG
$R_1$10$R_2$18-1-C5	TTCACATCATTT**CTAAGGTTCA**GGGGGAAAG
$R_1$10$R_2$18-2-C5	GAAAGGGGG**TGAACCTTAG**CGCAGGGTAGGTAGGTAGGGTCATAG
$R_1$10$R_2$20-1-C5	TTCACATCATTT**CTAAGGTTCA**GGGGGAAAGG
$R_1$10$R_2$20-2-C5	GGAAAGGGGG**TGAACCTTAG**CGCAGGGTAGGTAGGTAGGGTCATAG
$R_1$10$R_2$20-1-C5-mis	TTCACATCATTT**CTAAGGTTCA**GGGTGAAAGG
$R_1$10$R_2$20-2-C5-mis	GGAAAGTGGG**TGAACCTTAG**CGCAGGGTAGGTAGGTAGGGTCATAG
$R_1$10$R_2$22-1-C5	TTCACATCATTT**CTAAGGTTCA**GGGGGAAAGGG
$R_1$10$R_2$22-2-C5	GGGAAAGGGGG**TGAACCTTAG**CGCAGGGTAGGTAGGTAGGGTCATAG
$R_1$10$R_2$22-1-C5-mis	TTCACATCATTT**CTAAGGTTCA**GGGTGAAAGGG
$R_1$10$R_2$22-2-C5-mis	GGGAAAGTGGG**TGAACCTTAG**CGCAGGGTAGGTAGGTAGGGTCATAG
$R_{2\text{-imotif-C4}}$	CCCCTAACCCCTAACCCCTAACCCC
$R_1$8$R_2$16-1-C4	TTCACATCATTT**CTAAGGTT**GGGGTTAG
$R_1$8$R_2$16-2-C4	GTTAGGGG**AACCTTAG**CGCAGGGTAGGTAGGTAGGGTC

	ATAG
$R_1$8$R_2$18-1-C4	TTCACATCATTT**CTAAGGTT**GGGGTTAGG
$R_1$8$R_2$18-2-C4	GGTTAGGGG**AACCTTAG**CGCAGGGTAGGTAGGTAGGGTCATAG
$R_1$8$R_2$20-1-C4	TTCACATCATTT**CTAAGGTT**GGGGTTAGGG
$R_1$8$R_2$20-2-C4	GGGTTAGGGG**AACCTTAG**CGCAGGGTAGGTAGGTAGGGTCATAG
$R_1$8$R_2$20-1-C4-mis	TTCACATCATTT**CTAAGGTT**GGGTTTAGGG
$R_1$8$R_2$20-2-C4-mis	GGGTTATGGG**AACCTTAG**CGCAGGGTAGGTAGGTAGGGTCATAG
$R_1$8$R_2$22-1-C4	TTCACATCATTT**CTAAGGTT**GGGGTTAGGGG
$R_1$8$R_2$22-2-C4	GGGGTTAGGGG**AACCTTAG**CGCAGGGTAGGTAGGTAGGGTCATAG
$R_1$8$R_2$22-1-C4-mis	TTCACATCATTT**CTAAGGTT**GGGTTTAGGGG
$R_1$8$R_2$22-2-C4-mis	GGGGTTATGGG**AACCTTAG**CGCAGGGTAGGTAGGTAGGGTCATAG
$R_1$10$R_2$16-1-C4	TTCACATCATTT**CTAAGGTTCA**GGGGTTAG
$R_1$10$R_2$16-2-C4	GTTAGGGG**TGAACCTTAG**CGCAGGGTAGGTAGGTAGGGTCATAG
$R_1$10$R_2$18-1-C4	TTCACATCATTT**CTAAGGTTC**AGGGGTTAGG
$R_1$10$R_2$18-2-C4	GGTTAGGGG**TGAACCTTAG**CGCAGGGTAGGTAGGTAGGGTCATAG
$R_1$10$R_2$20-1-C4	TTCACATCATTT**CTAAGGTTCA**GGGGTTAGGG
$R_1$10$R_2$20-2-C4	GGGTTAGGGG**TGAACCTTAG**CGCAGGGTAGGTAGGTAGGGTCATAG
$R_1$10$R_2$20-1-C4-mis	TTCACATCATTT**CTAAGGTTCA**GGGTTTAGGG
$R_1$10$R_2$20-2-C4-mis	GGGTTATGGG**TGAACCTTAG**CGCAGGGTAGGTAGGTAGGGTCATAG
$R_1$10$R_2$22-1-C4	TTCACATCATTT**CTAAGGTTCA**GGGGTTAGGGG
$R_1$10$R_2$22-2-C4	GGGGTTAGGGG**TGAACCTTAG**CGCAGGGTAGGTAGGTAGGGTCATAG
$R_1$10$R_2$22-1-C4-mis	TTCACATCATTT**CTAAGGTTCA**GGGTTTAGGGG
$R_1$10$R_2$22-2-C4-mis	GGGGTTATGGG**TGAACCTTAG**CGCAGGGTAGGTAGGTAGGGTCATAG
$R_1$8$R_2$20-1-reset	/ROX/**CTAAGGTT**GGGGGGAGGG
$R_1$8$R_2$20-2-reset	GGGAGGGGGG**AACCTTAG**/BHQ2/
PRISM-1-3CA	TTCACATCATTT**CACCAAAGTCCACA**GGGGTTAGGGGAG
PRISM-2	GAGGGGTTTAGGGG**TGTGAACCTTAGTG**CGCAGGGTAGGTAGGTAGGGTCATAG

PRISM-3	CTCCCCTAACCCCTAACCCCTAACCCCTC
PRISM-4-3CA	TTCACATCATTT**CACCAAAGTCCACA**GGGGGGAGGGGGGAA
PRISM-5	AAGGGGGGAGTGGGG**TGTGAACCTTAGTG**CGCAGGGTAGGTAGGTAGGGTCATAG
PRISM-6	TCCCCCCTCCCCCCTCCCCCCTCCCCCCT
PRISM-7	TTCACATCATTT**CTAAGGTTCACA**GGGGGGAGGGGGGAA
PRISM-8-3CA	AAGGGGGGAGTGGGG**TGCGAACCCTAG**CGCAGGGTAGGTAGGTAGGGTCATAG
PRISM-9	TTCACATCATTT**CTAAGGTTCACA**GGGGGGAAAGGGGG
PRISM-10-2CA	GGGGGAAAGGGGGG**TGCGAACCCTAG**CGCAGGGTAGGTAGGTAGGGTCATAG
PRISM-11	CCCCCCTCCCCCCTCCCCCCTCCCCCC
PRISM-12	TTCACATCATTT**CTAAGTTCACA**GGGGGGAGGGGGGAA
PRISM-13-2CA	AAGGGGGGAGTGGGG**TGCGAACCTAG**CGCAGGGTAGGTAGGTAGGGTCATAG
PRISM-14-3CA	GGGGGAAAGTGGGG**TGCGAACCCTAG**CGCAGGGTAGGTAGGTAGGGTCATAG
PRISM$_4$-1-reset	/ROX/**CTAAGGTTCACA**GGGGGGAAAGGGGG
PRISM$_4$-2-reset	GGGGGAAAGGGGGG**TGCGAACCCTAG**/BHQ2/
Lettuce	CTTAGTAGGGATGATGCGGCAGTGGGCTTCGCAGT
Le-$R_1$10$R_2$10-1	**TTCTCTTAGT**AGGGATGATGCGGCAGTGGGCTCGCAGGCTCT
Le-$R_1$10$R_2$10-2	AGAGCCTGCGGGGG**ACTAAGAGAA**
Le-$R_1$10$R_2$8-1	**TTCTCTTAGT**AGGGATGATGCGGCAGTGGGCTCGCAGGCT
Le-$R_1$8$R_2$10-2	AGAGCCTGCGGGGG**ACTAAGAG**
Le-$R_1$10$R_2$6-1	**TTCTCTTAGT**AGGGATGATGCGGCAGTGGGCTCGCAGG
Le-$R_1$6$R_2$10-2	AGAGCCTGCGGGGG**ACTAAG**
Le-$R_1$10$R_2$4-1	**TTCTCTTAGT**AGGGATGATGCGGCAGTGGGCTCGCA
Le-$R_1$4$R_2$10-2	AGAGCCTGCGGGGG**ACTA**
Le-$R_1$10$R_2$2-1	**TTCTCTTAGT**AGGGATGATGCGGCAGTGGGCTCG
Le-$R_1$2$R_2$10-2	AGAGCCTGCGGGGG**AC**
Le-$R_1$10$R_2$0-1	**TTCTCTTAGT**AGGGATGATGCGGCAGTGGGCT
Le-$R_1$0$R_2$10-2	AGAGCCTGCGGGGG
Le-$R_1$10$R_2$10-2-2CA	AGAGCCTGCGGGGG**ACCAAAAGAA**
Le-$R_1$10$R_2$10$_{imotif}$-1	**TTCTCTTAGT**AGGGATGATGCGGCAGTGGGCT**AGGGGGGAGG**

Le-$R_1$10$R_2$10$_{imotif}$-2	CCCCCCTCCCCCCTCCCCCCTCCCCCCTGGGG**ACTAAGAGAA**
Le-$R_1$10$R_2$10$_{imotif}$-2-2CA	CCCCCCTCCCCCCTCCCCCCTCCCCCCTGGGG**ACCAAAAGAA**
$R_1$12$R_2$26-1-apt	*ATCAGGCTGGATGGTAGCTCGGTCGGGGTGGGTGGGTTGGCAAGTCTGAT*TTCACATCATTT**CTAAGGTTCACA**TCAACCGCTCTTT
$R_1$8$R_2$20-1-C6-apt	*ATCAGGCTGGATGGTAGCTCGGTCGGGGTGGGTGGGTTGGCAAGTCTGAT*TTCACATCATTT**CTAAGGTT**GGGGGGAGGG
PRISM-9-apt	*ATCAGGCTGGATGGTAGCTCGGTCGGGGTGGGTGGGTTGGCAAGTCTGAT*TTCACATCATTT**CTAAGGTTCACA**GGGGGGAAAGGGGG
PRISM-9-apt-random	*AACACCCTGGTTGGTAGCTCCCTCGGGGTGGGAGCCTTGGCAAGTCACAT*TTCACATCATTT**CTAAGGTTCACA**GGGGGGAAAGGGGG
Probe-Cy5	/Cy5/CTATGACCCTACCTACCTACCCTGCGATGATGTGAA
Probe-BHQ3	CGCAGGGTAGGTAGGTAGGGTCATAG/BHQ3/
Cr-28	GACCACCCCAAAAAUGAAGGGGACUAAAACCGCCACAAGCUCCAACUACCACAAGUUU
Cr-20	GACCACCCCAAAAAUGAAGGGGACUAAAACCGCCACAAGCUCCAACUACC
RNA-tar	AAACUUGUGGUAGUUGGAGCUUGUGGCG
B-5-16	AAACTTGTGGTAGTTGGAGCTTGTGGCGCATTTTTGGGGTGGTCTGGAAGGAGG
B-5-14	AAACTTGTGGTAGTTGGAGCTTGTGGCGTTTTTGGGGTGGTCTGGAAGGAGG
B-5-12	AAACTTGTGGTAGTTGGAGCTTGTGGCGTTTGGGGTGGTCTGGAAGGAGG
B-5-10	AAACTTGTGGTAGTTGGAGCTTGTGGCGTGGGGTGGTCTGGAAGGAGG
B-5-8	AAACTTGTGGTAGTTGGAGCTTGTGGCGGGGTGGTCTGGAAGGAGG
B-5-6	AAACTTGTGGTAGTTGGAGCTTGTGGCGGTGGTCTGGAAGGAGG
B-3-16	AAACTTGTGGTAGTTGGAGCTTGTGGCGGTTTTAGTCCCCTTCATGGAAGGAGG
B-3-14	AAACTTGTGGTAGTTGGAGCTTGTGGCGGTTTTAGTCCCCTTTGGAAGGAGG
B-3-12	AAACTTGTGGTAGTTGGAGCTTGTGGCGGTTTTAGTCCCCTGGAAGGAGG
B-3-10	AAACTTGTGGTAGTTGGAGCTTGTGGCGGTTTTAGTCCTGGAAGGAGG

B-3-8	AAACTTGTGGTAGTTGGAGCTTGTGGCGGTTTTAGTTGGAAGGAGG
B-3-6	AAACTTGTGGTAGTTGGAGCTTGTGGCGGTTTTATGGAAGGAGG
Tri-5-16	CCTCCTTCCAGACCACCCCAAAAATGCGCCACAAGCTCCAACTACCACAAGTTT
Tri-5-14	CCTCCTTCCAGACCACCCCAAAAACGCCACAAGCTCCAACTACCACAAGTTT
Tri-5-12	CCTCCTTCCAGACCACCCCAAACGCCACAAGCTCCAACTACCACAAGTTT
Tri-5-10	CCTCCTTCCAGACCACCCCACGCCACAAGCTCCAACTACCACAAGTTT
Tri-5-8	CCTCCTTCCAGACCACCCCGCCACAAGCTCCAACTACCACAAGTTT
Tri-5-6	CCTCCTTCCAGACCACCGCCACAAGCTCCAACTACCACAAGTTT
Tri-3-16	CCTCCTTCCATGAAGGGGACTAAAACCGCCACAAGCTCCAACTACCACAAGTTT
Tri-3-14	CCTCCTTCCAAAGGGGACTAAAACCGCCACAAGCTCCAACTACCACAAGTTT
Tri-3-12	CCTCCTTCCAGGGGACTAAAACCGCCACAAGCTCCAACTACCACAAGTTT
Tri-3-10	CCTCCTTCCAGGACTAAAACCGCCACAAGCTCCAACTACCACAAGTTT
Tri-3-8	CCTCCTTCCAACTAAAACCGCCACAAGCTCCAACTACCACAAGTTT
Tri-3-6	CCTCCTTCCATAAAACCGCCACAAGCTCCAACTACCACAAGTTT
B-5-14-imotif-probe	CCTCCTTCCAAATTACTTGGTAGTTGGAGCTTGTGGCGTTTTTGGGGTGGTCCCCCCCTCCCCCC
Tri-5-16-imotif-probe	CCCCCCTCCCCCCGACCACCCCAAAAACGCCACAAGCTCCAACTACCATACTCCCCCAGGTG
B-5-14-imotif	GGTAGTTGGAGCTTGTGGCGTTTTTGGGGTGGTCCCCCCCTCCCCCC
Tri-5-16-imotif	CCCCCCTCCCCCCGACCACCCCAAAAACGCCACAAGCTCCAACTACC
Cas-reporter	/ROX/UUUUU/BHQ2/
